# Is Emodin with Anticancer Effects Completely Innocent? Two Sides of the Coin

**DOI:** 10.3390/cancers13112733

**Published:** 2021-05-31

**Authors:** Esra Küpeli Akkol, Iffet Irem Tatlı, Gökçe Şeker Karatoprak, Osman Tuncay Ağar, Çiğdem Yücel, Eduardo Sobarzo-Sánchez, Raffaele Capasso

**Affiliations:** 1 Department of Pharmacognosy, Faculty of Pharmacy, Gazi University, Ankara 06330, Turkey; 2 Department of Pharmaceutical Botany, Faculty of Pharmacy, Hacettepe University, Ankara 06100, Turkey; itatli@hacettepe.edu.tr; 3 Department of Pharmacognosy, Faculty of Pharmacy, Erciyes University, Kayseri 38039, Turkey; gskaratoprak@erciyes.edu.tr; 4 Department of Pharmacognosy, Faculty of Pharmacy, Süleyman Demirel University, Isparta 32200, Turkey; tuncayagar@sdu.edu.tr; 5 Department of Pharmaceutical Technology, Faculty of Pharmacy, Erciyes University, Kayseri 38039, Turkey; cyucel@erciyes.edu.tr; 6 Instituto de Investigación y Postgrado, Facultad de Ciencias de la Salud, Universidad Central de Chile, Santiago 8330507, Chile; eduardo.sobarzo@ucentral.cl; 7 Department of Organic Chemistry, Faculty of Pharmacy, University of Santiago de Compostela, 15782 Santiago de Compostela, Spain; 8 Department of Agricultural Sciences, University of Naples Federico II, Via Università 100, 80055 Portici, Italy

**Keywords:** anthraquinone, apoptosis, cancer, drug discovery, emodin, nano-drug delivery

## Abstract

**Simple Summary:**

For cancer treatment, which is one of the most common and serious diseases of our day, miraculous secondary compounds of natural resources, which cannot be obtained even in the best chemical synthesis laboratories, are at the forefront. For this purpose, the anticancer activity of emodin, a natural anthraquinone derivative, on different cancer cell lines with different mechanisms of action is presented in pre-clinical studies. However, the activities of its synthetic derivatives were also evaluated due to their structural similarity to anthracyclines in general. In addition to its biological activity, qualitative and quantitative analyzes and information on high purification have been provided, and studies on the use of emodin in appropriate doses without causing side effects and toxicity have contributed to an impact on the scientific research community.

**Abstract:**

Many anticancer active compounds are known to have the capacity to destroy pathologically proliferating cancer cells in the body, as well as to destroy rapidly proliferating normal cells. Despite remarkable advances in cancer research over the past few decades, the inclusion of natural compounds in researches as potential drug candidates is becoming increasingly important. However, the perception that the natural is reliable is an issue that needs to be clarified. Among the various chemical classes of natural products, anthraquinones have many biological activities and have also been proven to exhibit a unique anticancer activity. Emodin, an anthraquinone derivative, is a natural compound found in the roots and rhizomes of many plants. The anticancer property of emodin, a broad-spectrum inhibitory agent of cancer cells, has been detailed in many biological pathways. In cancer cells, these molecular mechanisms consist of suppressing cell growth and proliferation through the attenuation of oncogenic growth signaling, such as protein kinase B (AKT), mitogen-activated protein kinase (MAPK), HER-2 tyrosine kinase, Wnt/-catenin, and phosphatidylinositol 3-kinase (PI3K). However, it is known that emodin, which shows toxicity to cancer cells, may cause kidney toxicity, hepatotoxicity, and reproductive toxicity especially at high doses and long-term use. At the same time, studies of emodin, which has poor oral bioavailability, to transform this disadvantage into an advantage with nano-carrier systems reveal that natural compounds are not always directly usable compounds. Consequently, this review aimed to shed light on the anti-proliferative and anti-carcinogenic properties of emodin, as well as its potential toxicities and the advantages of drug delivery systems on bioavailability.

## 1. Introduction

Emodin (1,3,8-trihydroxy-6-methyl-anthraquinone), a natural anthraquinone derivative found in the rhizomes and roots of mostly Rhamnaceae, Polygonaceae, Rubiaceae and Fabaceae plants, closely resembles the core of anthracyclines used in cancer therapy (carminomycin, daunorubicin, nogalamycin, doxorubicin) [1,2,3,4,5,6]. Emodin displays a variety of activities such as immunomodulatory, antibacterial, and anti-inflammatory properties [7,8,9]. Moreover, emodin has cytotoxic and growth-inhibitory effects against several types of tumor cells [10,11]. It displays these effects on different cell lines with different mechanisms. Increasing clinical findings show that inflammation causes cancer formation and inflammation-gene relationship is important in the initial phase. Almost all tumors are caused by inflammation, and it is known that if these inflammatory mediators cause cancer, the anti-inflammatory effect may be the first step in cancer treatment. Therefore, the mechanisms of anti-inflammatory activity of emodin have also been evaluated in anticancer effect. Studies have revealed that emodin suppresses cell growth and proliferation through the attenuation of oncogenic growth signaling, such as protein kinase B (AKT), mitogen-activated protein kinase (MAPK), HER-2 tyrosine kinase, Wnt/-catenin, and phosphatidylinositol 3-kinase (PI3K) that leads to apoptosis in several cancer cell types [12,13,14,15,16,17], and regulates cancer cell invasion and metastasis [18,19,20,21,22,23,24]. 

Emodin has an inhibitory effect on the proliferation of human lung squamous carcinoma CH27 cells [25], and human promyeloleukemic HL-60 cells, and stimulated apoptosis by provoking the caspase-3 cascade independently of reactive oxygen species (ROS) generating [16]. Emodin-caused apoptosis in human cervical cancer Bu25TK cells follows by poly (ADP-ribose) polymerase cleavage and the activation of caspase-9 [15] and another mechanism is probably related to the emodin-mediated capture at the G2/M phase of the cell cycle and differentiation causation [26]. Besides, emodin activates apoptosis in human hepatoma SK-HEP-1, PLC/PRF/5, and HepG2/C3A and cells by a p53-dependent pathway [27]. Furthermore, emodin improves arsenic trioxide-induced apoptosis by creating ROS and preventing survival signaling [28], and gene expression modification happens in HeLa cells through the redox-dependent improvement of arsenic cytotoxicity [29]. Emodin influences murine myelomonocytic leukemia WEHI-3 cells in vitro and increases phagocytosis in leukemic mice in vivo [30]. Emodin downregulates androgen receptors and inhibits the cellular growth of prostate cancer [23]. Emodin embarrasses the adhesion of human hepatocarcinoma (HepG2), human cervix epithelioid carcinoma (HeLa), and human breast cancer (MDA-MB-231) tumor cells by preventing lipid raft coalescence and impeding integrin gathering and focal adhesion complex (FAC) creation [21] and selectively prohibits the IL6-stimulated JAK2/STAT3 pathway, stimulates apoptosis in myeloma cells by the downregulation of myeloid cell leukemia 1 (Mcl-1) cells [20]. Also, it has been revealed that emodin might behave as a Janus-activated kinase 2 inhibitor and have cytotoxic effects against multiple myeloma in humans [20]. In the local ischemic myocardium, emodin brings guard from acute myocardial infarction over the prevention of apoptosis and inflammation [31].

Metal complexes of emodin were prepared and their anticancer activities were examined in previous studies. However, few researchers have tried to see if the metal-bound emodin can advance to its existing properties as an anticancer agent. In a cytotoxicity study, this complex was observed to be approximately three times more effective against five cancer cell lines compared to that of emodin alone [32]. Fascinatingly, numerous current studies have displayed that emodin might synergistically progress the anti-cancer efficiency of conventional chemotherapeutic drugs, such as cisplatin, etoposide, paclitaxel, and gemcitabine in pancreatic cancer [33], HER-2/neu-overexpressing lung cancer, and malignant melanoma without additional toxic effects [34,35,36,37].

This study debates the biological effects of emodin, its synthetic derivatives and its mechanisms that induce cell death in several human cancer cells’ types, both in vivo andin vitro. Investigation outcomes on emodin-stimulated cytotoxicity and its preventive properties against cancer in various body systems in addition to the mechanisms of antitumor included are defined.

## 2. Chemical Properties of Emodin

**Figure 1:**
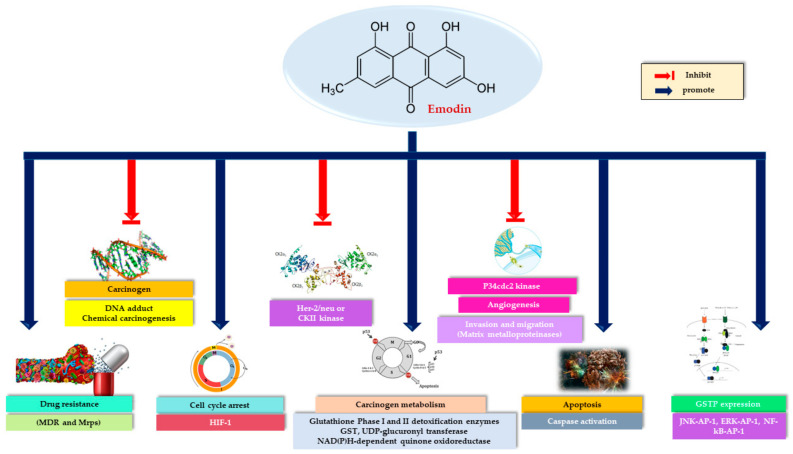
An overview of emodin’s anticancer efficacy.

Anthraquinones are numerous components derived from natural origin that are mutual in various organisms like plants, bacteria, fungi, and some animals. They are categorized as quinones and their derivatives are the largest natural quinone group. Plants are the resource of approximately 200 components of this class and are found in flowers, fruits, roots, and rhizomes. Mostof these compounds are derivedfrom the basic 9,10-anthracenedionestructure (Figure 1), a tricyclic aromatic organic compound. Anthraquinone glycosides are orange-brown colored polyphenolics and usually crystallinecompounds. In addition to being found in plants in their free form as aglycones, they are generally bound to sugar units such as glucose, rhamnose, glucorhamnose, or primeverose [38,39]. They are widespread species, particularly in the families of Rhamnaceae, Rubiaceae, Polygonaceae, Fabaceae, and Liliaceae. 

**Figure 2:**
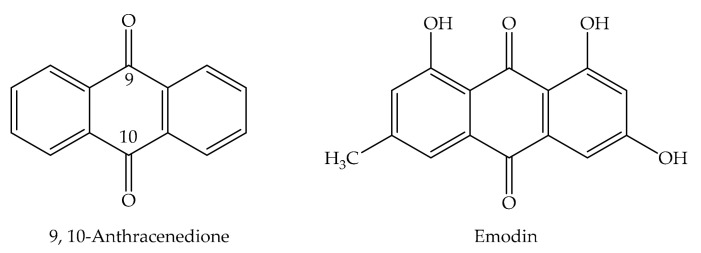
Chemical structures of 9,10-anthracenedione and emodin.

Emodin (Figure 2) is a chemical component from the anthraquinone family that can be obtained from rhubarb (*Rheum* sp.), aloe (*Aloe vera* L.), buckthorn (*Rhamnus frangula* L.), and Japanese knotweed (*Reynoutria japonica* Houtt. syn. *Polygonum cuspidatum* Sieb et Zucc. and *P. multiflorum* Thunb.). Emodin is significantly abundantin the roots of Chinese rhubarb (*Rheum palmatum* L.), knotweed, and knotgrass, as well as coffee weed. It is also obtained from several species of fungi, involving members of the genera *Aspergillus, Pestalotiopsis*, *Pyrenochaeta* and lichens. The commonname originates from *Rheum emodi* Wall. ex Meissn. the taxonomic synonym of Himalayan rhubarb (*Rheum australe* L.), and its synonyms includeemodol, frangula emodin, rum emodin, 3-methyl-1,6,8-trihydroxianthraquinone, Persian Berry Lake, and Schuttgelb [40].

### 2.1. Biosynthesis of Anthraquinones

Anthraquinones, aromatic polyketides, consist of an anthracene ring with carbonyl groups at the 9 and 10 positions. Anthracene-type compounds are orange, red-colored, and usually crystalline compounds. They are commonly found in plants of the families Rhamnaceae, Polygonaceae, Liliaceae, Rubiaceae, Fabaceae. Most of the drugs containing anthracene-type compounds are purgative; some of them, such as alizarin, are used as dyes in the textile industry. Three types of anthracene-derived compounds are found in plants: oxanthrones (enol forms), anthrones (enol forms), and anthraquinones. Among the three types the anthraquinone derivatives are the most durable, as the the others are oxidized [41]. Although their biosynthesis has not been fully explained, two main biosynthesis pathways have been identified that lead to the formation of anthraquinones in higher plants: (1) the polyketide pathway that provides hydroxylated di-ring polyketide anthraquinones through cyclization of the octa-*β*-ketoacyl-CoA chain formed by the addition of an acetyl-CoA to three malonyl-CoA units. As examples of anthraquinones produced by the polyketide pathway using type I or II polyketide synthases, emodin and chrysophanol may be mentioned, both found in rhubarb [42,43]; and (2) the shikimate or chorismate/o-succinylbenzoic acid pathway (a combination of shikimate and mevalonate/methyl-D-erythritol 4-phosphate pathways). This pathway is used to obtain 1,2-dihydroxyanthraquinones, and is that the biosynthesis of Rubia-type anthraquinones. In this way, Rubia-type anthraquinones’ rings A and B are originated from shikimic acid, -ketoglutarate via *o*-succinylbenzoate, while the C ring is originated from isopentenyl diphosphate, which is an essential structure for all isoprenoids. Obtained from *Rubia tinctorium*, alizarin has been derived from the shikimate pathway. Nevertheless, recent studies have focused on the fact that the biosynthesis of anthraquinones in plants occurs via a polyketide pathway [44,45,46,47].

### 2.2. Characterization of Anthraquinones

Anthraquinones can be described by their color responses in an alkaline medium, when converted into suitable phenolate ions. On the C-1 and C-8 carbons of 1,8-dihydroxyanthraquinone, the hydroxyl groups possess a similar acidity to that of carboxylic acids as they have similarscaffolds. Using weak alkalies like ammonium hydroxide, an alkalization reaction can be performed to obtain the phenolate ions. This reaction is defined as the Bornträger reaction and is utilized to properly identify anthraquinones in plant extracts [48].

Variations in coloration owing to reactions in an alkaline medium are appropriately utilized for the original structural characterization. Anthraquinones containing hydroxyls in positions 1 and 2, like 1,2-dihydroxyanthraquinones, produce a blue-violet color in a basic medium. In contrast, 1,8-substituted anthraquinones give a red color in basicmedia. Anthrones and dianthrones initially give a yellow color, and rapidly turn red as they are quickly oxidized to their corresponding anthraquinones [49]. 

### 2.3. Pharmacological Applications of Anthraquinones

Anthraquinones make up the largest natural quinone group, and more than 79 anthraquinones have been extracted from natural sourcesso far. The importance of this group of substancescan be understood by the use of various commercial derivatives in clinical therapy. Additionally, several medicinal herbs like *Aloe vera, Rheum rhabarbarum, Rhamnus purshiana* (cascara sagrada), *R. frangula,* and *Cassia acutifolia* with laxative effects; *R. tinctorum* which is utilized for bladder and kidney stones and *Hypericum perforatum*, which shows moderate antidepressant and sedative activityhave hydroxyanthraquinones as active ingredients [50,51]. 

The importance of emodin, a polyphenol anthraquinone derivative obtained from the roots and rhizomes of *Rheum palmatum* completely utilized in traditional Chinese medicinehas been emphasized. This compound is obtained primarily from three plant families: Fabaceae (*Cassia* spp.), Polygonaceae (*PolygonumRumex,* and *Rheum* spp.), and Rhamnaceae (*Rhamnus* and *Ventilago* spp.). Additionally, it was known to have antimutagenic, anti-inflammatory, and antimicrobial activities. It acts as a modulator of the vasomotor system and, among others, participates in metabolic and immunological processes [52].

Anthraquinones stand out for their extraordinary biological properties, including anticancer, antiarthritic, anti-inflammatory, antibacterial, antifungal, diuretic, and antimalarial effects. Additionally, some representatives of this class are currently clinically used and are commercially available. These compounds have uses in analytical chemistry and industrial processes for cellulose generation. They can be used as dyes, agricultural chemicals, and prototypes for the improvement of novel metabolites with biological activities. 1,2-Dihydroxyanthraquinone derivatives are used as dyestuffs, while 1,8-dihydroxy anthraquinone derivatives are used for their laxative effects [40].

**Figure 3:**
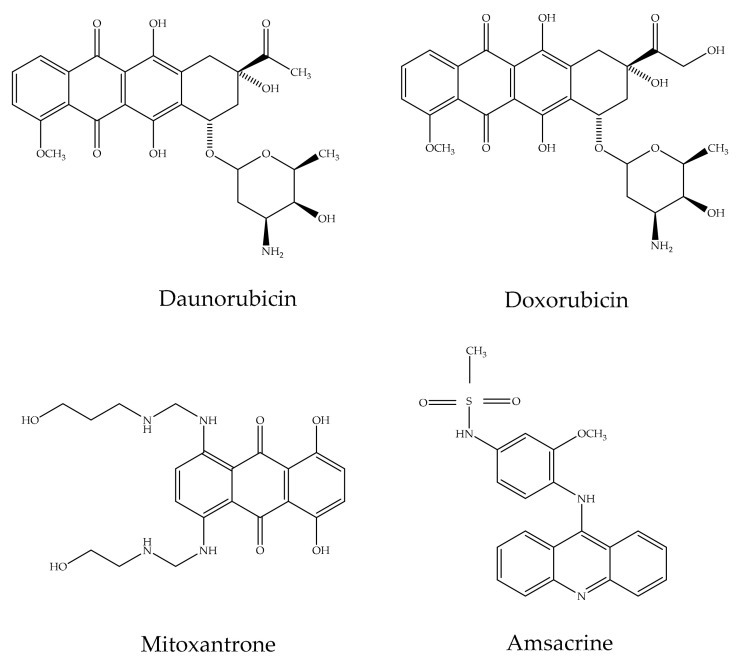
DNA-intercalating anthraquinones of clinical use.

Anthraquinones exhibit an important activity in the primary metabolism of plants by inhibiting energy transfer in the photosynthetic process by affecting the electron transport chain [53]. Additionally, these compounds are significant for their capacity to interrelate with DNA and block the enzyme topoisomerase II (topo II). Emodin possesses DNA-damaging features by stabilizing Topo-II-DNA covalent complexes and causes cell death through apoptosis. Currently, there are four DNA-intercalating components reproduced from anthraquinones authorized by the Food and Drug Administration (FDA) for clinical use: amsacrine, doxorubicin, daunorubicin, and mitoxantrone (Figure 3). These drugs, mitoxantrone (Onkotrone^®^) and daunorubicin (Daunoblastin^®^) represent some of the most potent cytostatics. They prevent tumor growth primarily by attaching to DNA and inhibiting topoisomerase II [40,54,55].

These compounds are considerable tools for treating inflammation, radiation, cancer, and diabetes-related injuries. Promising antidiabetic activities through the prevention of protein tyrosine phosphatase 1B (PTP1B) and α-glucosidase are shown by emodin and alaternin from *R. palmatum* and *Cassia obtusifolia.* Rhubarbis utilized in Traditional Chinese Medicine, and is an encouraging plant using with several anticancer activities by prevention of cell growth, cell cycle corruption, apoptosis induction, or antimetastatic actions. In addition to the numerous biological features of anthraquinones, their application in analytical chemistry is remarkable, often as potent chelating agents or chromophores.

Anthraquinones are used due to their capacity to undergo reversible responses given by the electrophilic character most of these components and their optical properties and intermolecular and intramolecular interactions like hydrogen bonds, which are directly linked to the structure and location of the substituents. They are used in photometry and fluorometry analyses and redox-activeand acid-base complexes [56,57].

## 3. Anticancer Activity of Emodin

### 3.1. Lung Cancer

The first studies evaluating the effects of emodin on lung cancer cell lines were conducted by Lee. The apoptosis mechanism of 50 µM emodin in CH27 cells wasconfirmed by morphological changes, sub-G1 formation, and disruption of focal adhesion kinase. While the modulation of endogenous Bcl-X (L) protein expression is not involved in this mechanism, the increase in the expression of cellular Bak and Bax proteins has been associated with apoptosis. It has also been stated that emodin increased the activation of caspase-3, caspase-8, and caspase-9 [25]. In a later study, the effect of emodin on apoptosis in CH27 and H460 cell lines was elucidated. It was determined that the effect leading to apoptosis in cells was internucleosomal DNA fragmentation, increase in cytochrome c (cyt c) at specified time intervals, and activation of caspase-3. However, there was an increase in the expression of PKCa and a decrease in the expression of PKCδ and ε was consistently seen in CH27 and H460 cells treated with emodin. While PKCδ and ε changes are known to play a significant role during apoptosis, the study focused on the possibility that the PKCδ catalytic fragment may be rapidly reduced to an undetectable smaller fragment [58]. The application of 50 µM emodin in A549 cells resulted in increased cyt c release and activation of caspase-2, -3, and -9. The inactivation of AKT and ERK, formation of reactive oxygen species (ROS), disruption of mitochondrial membrane potential (∆Ψm), reduction of mitochondrial B-cell lymphoma-2 (Bcl-2) and increase in mitochondrial Bax content were also important indicators in elucidating the mechanism. Highly redox-active molecules, quinones, may redox cycle with their semiquinone radicals and lead to the generation of ROS [59,60,61]. Therefore, in this study, emodin-based apoptosis has been demonstrated to be induced by ROS, which aregenerated from semiquinone [14]. Emodin treatment decreased the percentage of G0/G1 phase cells while increasing the percentage of both S and G2/M phase cells in NCI-H446 cell line. Emodin (20 μmol/L) increased caspase-3 activity. NACA, p8, and PQBP1 genes were up-regulated and B2M, HLA-E, and CD1D genes were downregulated. Emodin has been proven to play an active role in cell apoptosis, tumor metastasis, and chemotherapy resistance, as it affects the expression of genes involved in various cellular functions [62]. Chen et al. reported that emodin increased the cytotoxicity induced by gefitinib, a quinazoline derivative that may inhibit the phosphorylation of AKT, ERK1/2, and EGFR, in A549 and H1650 cells [63]. In the dose range of 2–10 μM, emodin did not cause ERK1/2 activation orchanges in mRNA and Rad51 protein levels, though, emodin treatment triggered Rad51 protein instability and caused a decrease in gefitinib-mediated phospho-ERK1/2 and Rad51 protein levels. In a different study, it was determined that emodin in A549 cells activates the ATM-p53-Bax signaling pathway created by reactive oxygen species and thus induces apoptosis. In the same study, pretreatment of cells with ascorbic acid prevented the induction of ROS by emodin and inhibited the upregulation of p53. It has been observed that the production of emodin-derived ROS leads to mitochondria-dependent apoptotic cell death [64]. Emodin-induced cytotoxicity has been demonstrated in the lung cancer cell lines H1650, A549, H520, and H1703 through inactivation of ERK1/2 and down-regulation of ERCC1, and Rad51. According to the results, emodin has a certain effect on ERCC1 expression in lung cancer cell lines and has been reported to increase the cytotoxic effects induced by siRNA transfection [65]. It was determined by Ko et al. in a different study that emodin increased capecitabine-induced cytotoxicity by affecting Rad51, ERCC1, and thymidine phosphorylase (TP) expression in the same cell lines. These cytotoxic effects are associated with ERK1/2 inactivation and decreased ERCC1 and Rad51 protein levels. Also, emodin increased mRNA and protein expression of TP in capecitabine-treated cell lines [66]. In the combination study of cisplatin and emodin, the effects on H1703 and H520 cells were evaluated. Similar mechanisms previously mentioned, were also observed in this study. Accordingly, emodin increased the instability of the ERCC1 protein, decreased cisplatin-induced ERK1/2 activation and ERCC1 protein induction [67]. Mitomycin C-sensitive phosphorylated ERK1/2 and Rad51 levels decreased because of exposure of H1703 or A549 cell lines to emodin. Emodin has been observed to significantly lower the levels of mitomycin C-derived Rad51 mRNA and protein by decreasing the stability of the Rad51 mRNA and protein. Emodin caused inhibition on the proliferation of A549 and SK-MES cells in a concentration-dependent manner. The protein and mRNA expression of ERCC1 and Rad51 also decreased significantly with the administered doses of 1–40 μmol/L (SK-MES), 70 μmol/L (A549). Vacuolar degeneration has also been observed in these cell lines [68]. The detailed mechanism of emodin involved in the regulation of CXC chemokine receptor-4 (CXCR4) gene expression, which affects cellular migration and invasion in lung cancer cells, was first studied by Ok et al. Emodin at a concentration of 100 μM considerably suppressed the expression of CXCR4 and also the expression of human epidermal growth factor receptor 2 (HER2) [69]. It has been previously reported that emodin can sensitize non-small cell lung cancer cells overexpressing HER-2/neu to chemotherapeutic drugs such as doxorubicin, VP16, and cisplatin [37]. Emodin has been reported to potently inhibit four members (BR1, AKR7A2, AKR1B10, AKR1A1) of the six most human anthracycline reductase in A549 cells and sensitized cancer cells toward daunorubicin [70].

In A549, PC9, H1299, H1650, and H1975 cells, emodin inhibited cell growth and caused an increase in the phosphorylation of AMP-activated protein kinase α subunit (AMPKα) and MAPK extracellular signaling-regulated kinase (ERK1/2). It has been determined that integrin-linked kinase (ILK) protein expression inhibited by emodin is through activation of AMP-activated protein kinase [71]. A later study by Tang et al. found that emodin triggers cell cycle arrest in the G2/M phase in A549 cells and causes invasion inhibition. Increased PPARγ protein and luciferase reporter activity were observed. In addition, IGFBP1 mRNA increased protein and promoter activity through activation of PPARγ [72]. It was determined that emodin had no noticeable effect on A549 cells at concentrations less than 1 µM and caused a slight decrease in cell viability at a concentration of 10 µM and prevented ATP-induced increases in P2X7 cancer cell invasion and related changes within the cell [73]. A549 and H1299 cells were treated with emodin at concentrations of 20, 40, 60, and 80 µmol/L, the results indicated that viability was significantly reduced at 60 and 80 µmol/L. The apoptotic rate of cancer cells and cleaved caspase-3 expression were significantly increased at increasing emodin concentrations. The increased TRIB3 expression, which is an important cell regulator of apoptosis, by emodin has shed light on the apoptotic mechanism. Then, the effect of emodin on the NF-κB pathway was investigated to fully elucidate the mechanism. When PDTC, a selective NF-κB inhibitor, was used in emodin-treated cell lines, it was found that emodin could not increase apoptosis. To examine the in vivo effects of emodin on tumor development, subcutaneous tumors were created by implanting A549 cells in BALB/c nu/nu mice. It was reflected in the results that the emodin significantly reduced tumor volume [74].

Emodin isolated from an endophytic fungus was assessed in A549 cells. Isolated emodin (30–100 µg/mL) induced cell death by arresting cells in the G1 and G2/M phases in the A549 cell line. Loss of ∆Ψm enhances the release of cyt c from mitochondria and also triggers other apoptotic factors, by activating the mitochondrial permeability transition pore. A significant decrease in the ∆Ψm was observed after 48 h emodin treatment [75]. Emodin’s effect on A549 cells was elucidated by a different mechanism by Haque et al. Since the loss of p53 function by mutation is a common cause of human cancers, the effect of emodin on p53 aggregation has been evaluated. Removal of p53 protein aggregates after emodin administration in A549 cells increases the level of autophagy [76]. It was found by Chen et al. that the mixture of emodin and paclitaxel synergistically inhibited the proliferation of A549 cells. The apoptotic mechanism of the combination was expressed as the increase of Bax and active caspase 3 expressions and the decrease of p-Akt, p-ERK, and Bcl-2 levels. In the in vivo part of the study, the effects of the combination on apoptosis and Akt signaling pathway in A549 tumor tissues were summarized as a decrease in p-Akt, an increase in active caspase -3 [77].

Li et al. have offereda different perspective on emodin’s effect on lung cancer [78]. Anticoagulation and anti-cancer effects were evaluated in a urethane-induced lung cancer model in the study. Emodin treatment exhibited a reduction in lung carcinogenesis and hypercoagulation in the alveolar space accompanied by N2 neutrophils (CD66b+). Cit-H3 and PAD4 were decreased in the lung compartments and tumor growth was inhibited in the Lewis lung cancer (LLC) allograft model [78]. Cisplatin-emodin combination increased DNA damage, decreased Pgp expression in A549 and H460 cell lines, but no effect was observed on MRP1 expression [79]. Emodin reduced the viability of lung cancer cell lines A549, H520, H1975, H1299, and H460 in a dose-dependent manner in the concentration range of 1–30 µM. It suppressed the secretion of Hyaluronan (HA), a molecule associated with malignancy in the cancer microenvironment, in cell lines except for H460 cells. Emodin significantly augmented cells in the G1/G0 phase and reduced cells in the G2/M and S phases in the A549 cell line. In addition, while decreasing the expression of cyclin A and cyclin B in the same cell line, it increased the levels of cyclin C, cyclin D, and cyclin E. These cyclin proteins are not altered by emodin exposure when HAS2 is deactivated, suggesting that emodin regulates HAS2, affecting the cell cycle [80].

Redox protective phosphatase, Mut T homolog 1 (MTH1) expression, which performs enzymatic conversion of oxidized nucleotides to corresponding monophosphates, is upregulated in a large number of cancers. For this reason, the molecular screening result for MTH1 active site binders, emodin was identified as a lead compound for MTH1 active site functional inhibition. Expression and activation of cell cycle-related proteins were investigated, as binding emodin and MTH1 have been shown to cause cell cycle arrest in the G2/M and cause a decrease in the S phase in NCI-H-520, NCI-H-460, and A-549 cells. While an increase in the number of apoptotic cells was observed at the end of 72 h in all three cell lines, the highest rate was found in NCI-H-520 cells with approximately 63.9%. In NCI-H-520 cells treated with 50μM emodin, the expression of Bax (pro-apoptotic protein) and survivin increased and anti-apoptotic protein Bcl-2 decreased. Increased P-21 protein levels, cleaved-caspase-3, and cleaved-PARP levels, and decreased p-65 NFκβ protein levels were also observed. The cell migration was suppressed ~70% at 48 h in NCI-H-520, which was confirmed by reduced integrin β1 and vimentin protein expression levels. It has been reported that triggering ROS formation by emodin causes mitochondrial membrane depolarization and subsequent induction of apoptosis in non-small-cell lung carcinoma cells [81]. The in vitro biochemical effects of emodin in lung cancer cell lines are summarized in Table 1.

In various lung cancer cell lines, emodin shows activity by inhibiting cancer cell proliferation, increasing ROS levels, chemotherapeutic sensitivity and inducing apoptosis. Emodin’s anti-cancer potential in lung cancers has shown great advances and its anti-tumor activity has been demonstrated through a variety of mechanisms. Apoptosis mechanisms consist of inhibition of protein kinases CK2, increase in the phosphorylation of AMPKα, and ERK1/2, increase in the activation of caspase-2,-3, -8, and -9, cyt c release, formation of ROS, disruption of ∆Ψm, reduction of Bcl-2 and increase in mitochondrial Bax content, overexpression in HER-2/*neu.* Combined therapy studies with gefitinib, cisplatin, paclitaxel, daunorubicin and capecitabine have revealed promising results.Considering the studies conducted, the anticancer activity of emodin has been studied in detail in lung cancer cell lines and its effect on many pathways has been proven.However, although it is known that there are still unexplained mechanisms, it is obvious that the continuation of these studies without slowing down will increase the clinical value of emodin.

### 3.2. Breast Cancer

**Figure 4:**
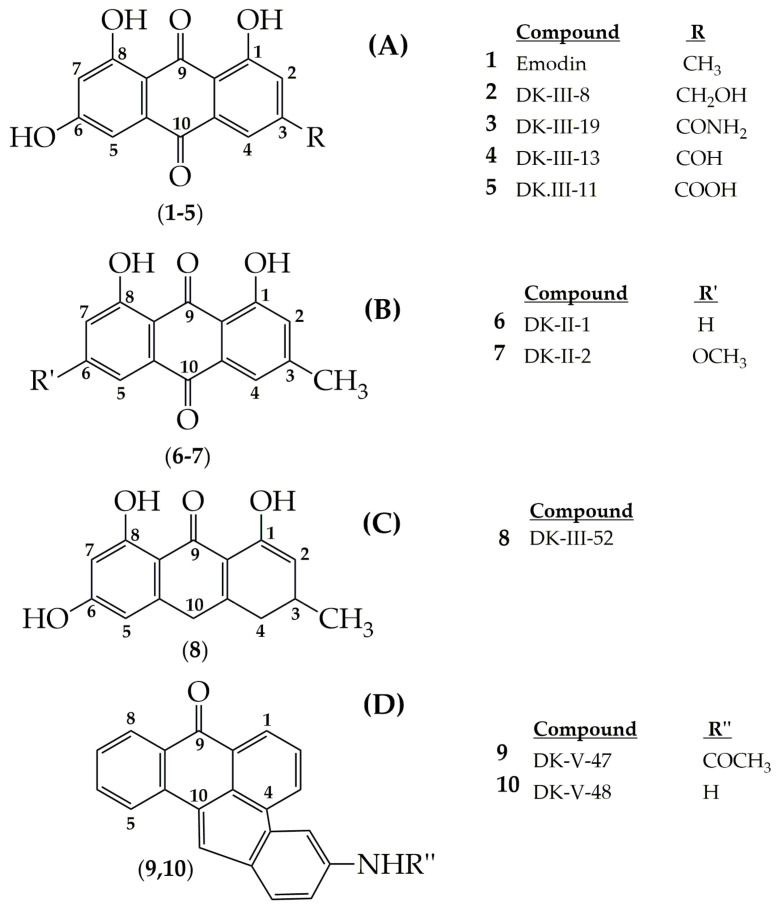
Chemical structures of emodin and its synthesizedderivatives.

Overexpression of the HER-2/neu proto-oncogene and occurs in about 30% of breast cancers and is associated with reduced survival of breast cancer patients. For this reason, emodin, a protein tyrosine kinase inhibitor, was examined in detail in the first studies by Zhang et al. [17]. In the first study, emodin caused tyrosine hypophosphorylation pl85neu in HER-2/neu overexpressing MDA-MB453, BT-483, and AU-565 cells by suppressing the autophosphorylation and transphosphorylation activities of HER-2/neu tyrosine kinase. At a concentration of 40 µm, emodin inhibited the growth of AU-565, BT-483, and MDA-MB453 cells by 68%, 72%, and 84%, respectively. However, it could suppress 37% and 23% growth in MDA-MB 231 and MCF-7 cells. In the immortalized breast cell line (HBL-100), even at a concentration of 80 µM, it had little effect on viability. MDA-MB453 and AU56 cells showed a flat morphology with larger nuclei after emodin administration and an increase in the cytoplasm were detected but no morphological change was observed in MCF-7 [17]. In the next study, the effects of emodin and ninederivatives on MDA-MB453, MCF-7, and B104-1-1 cells were evaluated. The nine synthesized emodin derivatives (Compounds 2-10) are shown in Figure 4. One of the emodin derivatives, 10-(4-acetamidobenzylidene)-9-anthrone, was found to be more potent than emodin in suppressing the tyrosine phosphorylation of p185neu and inhibiting the proliferation and transformation of HER-2/neu-overexpressing human breast cancer cells. When the gelatinase collagenase IV activity was evaluated in HER-2/neu-transformed 3T3 cells, (B104-1-1 cells), it was determined that a 10 µM dose of potent derivative inhibits the gelatinolytic activity better than at 40 µM concentrated emodin. Emodin (40 µM) and its potent derivative (20 µM) induce cell cycle arrest at G0/G1 phase in MDA-MB 453. It was concluded that 10-(4-acetamidobenzylidene)-9-anthrone derivative is more pronounced than emodin, by the way, suppressing transformation phenotypes of activated HER-2/neu-transformed 3T3 cells both anchorage dependent and -independent growth, and metastasis-associated properties [84].

In the in vivo study by Zhang et al. [36] using the same mechanism, emodin (40 mg/kg bw, *i.p*) was found to suppress tumor growth and prolong the survival of mice in mice carrying HER-2/neu-overexpressing human breast cancer cells (MDA-MB-361). However, emodin could not significantly inhibit the growth of MDA-MB-231 tumors or ensure the long-term survival of mice with such tumors compared to placebo. In the same study, it was determined that the combination of emodin-paclitaxel inhibited the anchorage-dependent and independent growth of MDA-MB-361 and BT-474 cells (breast cancer cells overexpressing HER-2/neu). However, the combination therapy showed no significant antiproliferative effect in the MDA-MB-231 and MDA-MB-435 cells (low levels of HER-2/neu). The combination of emodin and paclitaxel displayed more significant results in in vivo experiments. The inhibitory effect of emodin followed by paclitaxel injection on tumor growth was found to be highly effective. The combination of emodin- paclitaxel application gave significantly better results than single therapy applications. The inhibition of the phosphorylation of HER-2/neu tyrosine kinase was confirmed when in vivo experimental results were evaluated by both histopathological and western blot analysis [36]. A doxorubicin and emodin combination was evaluated both in vitro and in vivo by Li et al. more recently. According to in vitro results, it was determined that 48 h application of emodin at a concentration of 110 µM increased the sensitivity of MDA-MB-231 and MCF-7 cells to doxorubicin (5 µM). Combination treatment increased the γH2Ax expression and the DNA damage and resulted in decreased expression of the AKT1, p53, PARP1, RAD51, and XRCC1 signaling pathway. According to the in vitro test results, it was determined that tumor size, weight, and KI67 expression decreased [80]. However, in contrast to these studies, a different combined therapy study showed that emodin reduced the effectiveness of tamoxifen in ZR-75-1 and MCF-7 cells. The antagonistic effect of the combination of emodin (60 µM) and tamoxifen (4 µM) has been associated with the up-regulation of cyclin D1 and p-ERK [85].

In the MDA-MB-231cell line, the mechanism of emodin (10–40 µM) was described as inhibiting the TPA-stimulated MMP-9 activity, reducing the transcriptional activity of activator protein-1 (AP-1), nuclear factor kappa B (NF-κB) [86]. BCap-37 cells when incubated with 50 µM emodin significantly increased the percentage of cells in the sub-G0/1 phase compared to the 20 µM incubation. Apoptotic features such as cellular morphological change, chromatin condensation, and membrane swelling were determined in cells treated with emodin. When important proteins associated with apoptosis were examined, it was observed that Bcl-2 decreased, Bax level, and cytosolic cyt c level increased [87]. Huang et al. later examined 458 genes associated with apoptosis with cDNA microarray in the same cell line. 50 µM emodin treatment caused an up-regulation of cyclin-dependent kinase inhibitor 1 (P21) and down-regulation of insulin-like growth factor 2 (IGF-2). Also, gene expression of p53 and caspase 3 was induced. These results indicate that the emodin’s cellular apoptosis in breast cancer was related to the disruption of the mitochondrial-signaling pathway [88]. In another study, it was found that emodin also caused apoptosis in SKBR3 cells via caspases. Caspase -3, -8, and -9 mRNA levels increased in SKBR3 cells after administration of emodin at 25 and 50 μM concentration for 48 h. A significant increase in Bax and reduction in Bcl 2 were also observed [89].

**Figure 5:**
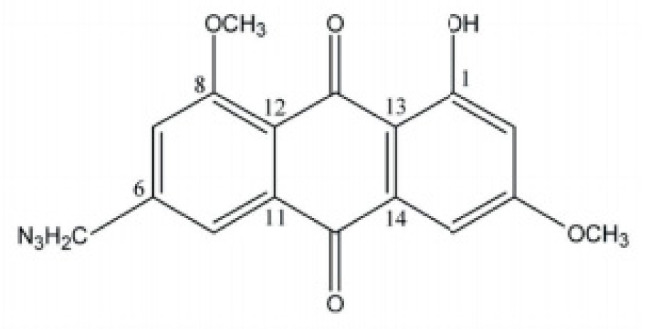
Chemical structure of emodin azide methyl anthraquinone derivative.

In the study of Yan et al., MDA-MB-453, MCF-7/ADR, and MCF-7 cells were used to evaluate the effect of emodin azide methyl anthraquinone derivative (Figure 5) on Her2/neu protein [90]. This emodin derivative at a concentration of 2.3 to 9.2 µg/mL strongly lowered the Her2/neu protein and also inhibited the downstream MAPK and PI3K-Akt signaling pathway by inhibiting p-Akt, and p-ERK1/2 in the MDA-MB-453 cell line [90]. Yan et al. investigated the effects of the same emodin derivative on the cell cycle and related proteins in MDA-MB-453 cells in their later study. This potent emodin derivative (2.30–9.20 μM) arrested cells in the G0/G1 phase after 24 h and inhibited the expressions of Cyclin D1, c/Myc, CDK4, and p-Rb [91]. 

The effects of emodin on the resistance of MCF-7/Adr cells, which are 11-fold resistant to cisplatin and 21-fold to adriamycin, investigated by Fu et al. Emodin at a concentration of 10 µg/mL was found to reduce this resistance, with a decrease from 21-fold to 2.86-fold for adriamycin and 11-fold to 1.79-fold for cisplatin. Emodin at a concentration of 20 μg/mL in MCF-7/Adr cells significantly decreased excision repair cross complementation group 1 (ERCC1) expression, which can lead to multi-drug resistance to chemotherapy in cancer treatment [92]. Emodin (25 μM) could not support HSP90/ERα decomposition and estrogen receptor α (ER α) ubiquitination in MCF-7 and MDA-MB-453 cells. Although emodin could trigger cytosolic ERα degradation, it primarily effected nuclear ERα distribution, similar to the effect of estrogen when protein degradation was inhibited [93]. In the study examining the effect of emodin on proliferation and its mechanism leading to apoptosis, the growth inhibition IC50 value was found to be 7.22 µg/mL. The inhibition effect on the colony-forming ability of MCF-7 cells was indicated as IC_50_ = 7.60 µg/mL. Single-stranded DNA breaking, DNA fragmentation, up-regulation of Fas ligand (FASL) gene expression, and distinct features of apoptosis, such as down-regulation of the expression of CCND1, C-MYC, and MCL1 were observed after emodin (35 µM) treatment [94]. 

Studies have revealed that the P2X7Rs play a significant role in increasing the invasive phenotype of aggressive MDA-MB-435 s human breast cancer cells by mediating the release of active cathepsins in the extracellular microenvironment. In a study evaluating the potential antagonism of emodin on human P2X7R, the effect on intracellular Ca2+ concentration (a dependent increase on human P2X7R) in MDA-MB-435s cancer cells was examined. ATP caused a sustained increase in [Ca2+] in MDA-MB-435 s cells, but this effect disappeared when the cells were pretreated with a P2X7R selective antagonist. Similarly, emodin could inhibit the ATP-induced increase in [Ca2+] depending on the dose in the concentration range of 1 and 10 µM. Simultaneously, gelatinolysis of the periocular matrix was significantly increased in ATP-stimulated cells, but this effect was reversed in the presence of 1 µM emodin. In this study, consistent results were obtained, showing that emodin prevents human cancer cell invasion [73].

It has been shown in the study by Sui et al. that emodin can inhibit estrogen-induced MCF-7 cell proliferation and anti-apoptosis effects (20 and 40 µM). This anthraquinone arrested the cell cycle in the G0/G1 phase and blocked the effect of estrogen on ERα expression and transcriptional activity. The ERα genomic pathway was mediated by down-regulation of cyclin D1 and Bcl-2 protein expression, and the non-genomic pathway by decreased PI3K/Akt protein expression [95]. 

Since emodin has a strong anti-inflammatory effect [96,97,98], there are also studies that associate this effect with cancer. Macrophages treated with emodin at concentrations of 10 µM and 30 µM were applied to breast cancer cells and allowed to interact with and adhere to each other. When adherent macrophages were examined and counted, it was determined that there was a significant difference between the groups, especially in the 30 µM mm dose of the emodin group, there was less adhesion between macrophages and cancer cells [99]. In the in vivo experiment for the antimetastatic effect of emodin, tumors were formed with 4T1 and EO771 breast cancer cells. 40 mg/kg emodin was injected once a day intraperitoneally. Emodin has been reported to significantly suppress 4T1 lung metastatic colonization in mice. In the transwell migration assay, the 4T1 cell-conditioned media was found to cause a sharp increase in macrophage migration, but emodin was shown to significantly inhibit this in a dose-dependent manner, especially at a dose of 100 µM [97]. A similar study was conducted by Iwanowycz et al. using EO771 and 4T1 breast cancer cells. Daily administration of 40 mg/kg emodin (*i.p*) resulted in a significant reduction in primary tumor growth, tumor size, and tumor weight in both EO771 and 4T1 tumor models. In the EO771 tumor model, it was also determined that emodin caused a significant decrease in the number of tumor-infiltrating macrophages. It was shown that tumor-associated macrophages in emodin-treated mice had significantly lower Arg1 and CD206 marker expression, but significantly higher iNOS expression. Emodin significantly reduced the number of macrophages also in 4T1 tumors 26 days after implantation and caused a significant decrease in the fraction of macrophages positive for the transcription factors pSTAT6 and C/EBPβ. When isolated tumor-infiltrating macrophages from 4T1 tumors were analyzed, reduced expression of interferon regulatory factor 4 (IRF4), which plays a significant role in macrophage M2 activation, and relatively increased H3K27M3 (Histone 3 Lysine 27 Tri-methylation) levels and increased T cell activation were found in breast tumors. Suppression of angiogenesis has been associated with increased T cell activation of emodin. It has also been determined that pretreatment of macrophages or tumor cells with emodin in vitro significantly inhibits the adherence of macrophages to the monolayer of tumor cells [100]. In the next study of the same team, tumorswerecreated with 4T1 cells in mice. Emodin treatment (40 mg/kg, i.p) did not alter the tumor size and weight, but suppressed macrophage infiltration in the tumors (particularly CD206+ M2-like macrophages), increased CD4+ and CD8+ T cells, and decreased the expression of Vimentin and MMP9. According to in vitro results, emodin decreased TGF- β1 production in breast cancer cells and macrophages and inhibited the formation of EMT and CSC [101]. For the same purpose, Song et al. used MDA-MB-231 cells in the tumor model. 40 mg/mL emodin (*p.o*) for 21 days treated group of mice, emodin reduced CC chemokine ligand 5 (CCL5) level, tumor growth, lung, and liver metastasis. In the in vitro part of the experimental study, when MDA-MB-231 and MDA-MB-453 cells were incubated in the supernatant of adipocytes, it was observed that emodin was more effective against resistance formed than epirubicin, which was used as a positive control. In the transwell and wound healing experiments, 25 μM emodin treatment resulted in the inhibition of triple-negative breast cancer cell migration and invasion. Decreased CCL5 levels were also detected in the cell culture supernatants, indicating that emodin has the ability to inhibit proliferation and migration. According to the in vitro results, it was determined that emodin inhibits the phosphorylation of AKT, activates GSK3β, downregulates the expression of β-catenin and mesenchymal related proteins vimentin and snail, and increases the expression of epithelial related protein E-cadherin [102].

Zu et al. evaluated the effect of emodin on Notch-regulated ankyrin repeat protein (NRARP) since NRARP was found to stimulate cell proliferation and overexpression in breast cancer. In MCF-7 cells, for 48 h emodin (20 μM) treatment with 5-fluorouracil (5-FU, 40 μM) showed a strong decrease in cell viability. Up-regulation of p21, p16, p27 protein, down-regulation of E2F1 and NRARP protein, enhanced ROS formation, and induced cellular senescence were observed after combination treatment [103]. Salt-inducible kinases 3 (SIK3) belonging to the AMPK-related kinase family play a role in the regulation of cell metabolism, remodeling of cell polarity, and epithelial-mesenchymal transition. The combination activity of berberine and emodin on this kinase was evaluated on MCF-10A, MCF-7, and MDA-MB-231 cells, as high SIK3 expression in breast cancer cells contributes to tumor formation. Combined therapy in breast cancer cells by suppressing SIK3 activity, it resulted in decreased cell growth, cell cycle arrest (G0/G1), and apoptosis, but these effects were not observed in healthy mammary epithelial cells [104].

In a different study evaluating the combination of two natural compounds, thymoquinone and emodin, MCF-7, T47D (p53 wild type) and MDA MB 231 and MDA MB 468 (mutant-p53) cell lines were first used for cytotoxic effects. The cytotoxic effect was observed stronger in MCF-7 and T47D cells in combination therapy, while no change in effect was observed in mutant-p53 cells. Therefore, in the next part of the study, MCF-7 and T47D cells were used. Combined treatment with emodin 10 µg/mL and thymoquinone 2 µg/mL resulted in approximately 50% cell arrest in the sub G0/G1 phase in MCF7 cells and inhibition of colony formation. Apoptotic cell death increased to 63.60% because of the same combination therapy compared to administration alone. An increase in p53, Bax, and cleaved caspase 3 expression levels, a decrease in Bcl-2 protein, induction of ROS formation, and Cyt C release were also observed in the MCF 7 cell line. Apoptotic cell death was determined as 55% in T47D cells. Combined treatment with emodin 5 µg/mL and thymoquinone 1 µg/mL inhibited cell migration and FAK, pFAK, and integrin β1 proteins were down-regulated in both cell lines. In ex vivo CAM model combination treatment also inhibited tumor growth and metastasis [105]. Again, in a study conducted in MCF7 cells, emodin was reported to cause 28.89–47.64% apoptotic death in the concentration range of 25–100 µmol/L. Emodin administered at increasing concentrations caused an increase in CYP1A1 expression. According to the results of the study, emodin shows its effect on cell proliferation by regulating the expression of AhR and CYP1A1 proteins [106]. In vitro biochemical effects of emodin in breast cancer cell lines are summarized in Table 2.

Strong efficacy has been observed in almost all studies examining the potency of emodin in in vitro studies with breast cancer cell lines and in vivo tumor models. While positive results were obtained in combination studies with paclitaxel and doxorubicin, an antagonistic effect was observed in the tamoxifen combination. Simultaneously, the combinations of berberine and thymoquinone showed synergistic effects. In general, the mechanisms underlying the anticancer effect have been reported as follows: Increased ROS generation, Bax level, and cytosolic cyt c level, decreased Bcl-2 level, inhibition of AKT phosphorylation with activation of caspases, inhibition of Her2/neu phosphorylation to block Her-2/neu/PI-3K signaling pathway.

### 3.3. Gastric Cancer

Gastric cancer (GC) is known as one of the most common cancer diseases worldwide. Recent studies show that emodin may be effective on GC. In the study conducted by Chihara et al., it was recorded that after 0.05 mM emodin treatment, significant inhibition of proliferation on human gastric cancer cell line (MKN45) occurred 24 and 48 h later. The anticancer activity of emodin occurred due to arrest in the G0/G1 and G2/M phases [107]. The use of emodin in combination with cisplatin (CDDP), a drug currently used for gastric cancer, has been observed to increase the apoptotic effect on SNU-5 cells. The reasons for the antitumoral effect of emodin were investigated using MTT, Annexin V-Binding test with flow cytometry, and transmission electron microscopy. With the combination of 25 μM emodin + 3.0 μM CDDP, the inhibitory effect on growth was synergistically increased significantly. With the concomitant use of the two compounds, apoptosis and cell cycle arrest were significantly increased compared to single uses. Transmission and fluorescence electron microscopy investigations disclosed that the concurrent treatment with emodin and CDDP caused changes in cell morphology and apoptosis induction. As a result, combination treatment with emodin may produce much more effective results in patients with gastric cancer instead of using a single drug [108]. Emodin was found to have an antiproliferative effect on SGC-7901 cells characterized by the down-regulation of PRL-3. Early apoptosis rates increased depending on the concentration after emodin application. Additionally, real-time quantitative PCR analysis results exhibited a significant reduction of PRL-3 mRNA. As a result of Western blot analysis, it was observed that PRL-3 protein expression was down-regulated. It has been recorded that PRL-3 can be a beneficial therapeutic marker for gastric cancer [109]. It is very important to control drug release in the stomach and to design new vectors for this to reduce the systemic adverse effects of stomach diseases as much as possible and to determine a better treatment method. For this reason, chitosan-coated nanomicelles, a gastro-retentive drug delivery system, were developed and characterized by Chen et al. The study compared the cytotoxic effects of emodin suspensions, emodin-loaded nanomicelles, and empty nanomicelles on human gastric carcinoma SGC-7901 using the MTT method. While no cytotoxicity was observed in empty nanomicelles, the best antitumoral effect was observed in emodin-loaded nanomicelles. In addition, the local release was aimed at preparing nanomicelles-loaded floating mucoadhesive beads (NFM-Beads). Nanomicelles and NFM-beads formation was observed by the FT-IR spectroscopy method. The residence time of NFM-beads after dosing was measured as 8 h. These results show that emodin released by the NFM-beads system can be much more effective on stomach cancer [77]. In a similar study by Jiang et al. emodin loaded stearic acid-g-chitosan oligosaccharide (CSO-SA/EMO) was prepared and characterized. The prepared nanomicelles were tested on gastric cancer cell lines BGC823 and MGC803. Nanomicelles were applied for 12 h and accumulated abundantly in mice. As a result, emodin given loaded with nanomicelles showed a significant antitumoral effect in the cell lines tested [110]. The role of emodin in gastric cancer treatment has been investigated on in vitro models, and its mechanisms of action, in general, are reported as follows: Inhibition of proliferation, arrest in the G0 / G1 and G2/M phases, increase the apoptotic effect on cells, down-regulation of PRL-3 mRNA. The in vitro effects of emodin in gastric cancer cell lines are summarized in Table 3.

### 3.4. Pancreatic Cancer

The recent studies presented that in comparison with the control emodin stimulated significant growth prevention and apoptosis in the pancreatic cancer cell line, preventing migration and incursion of SW1990 cells in a dose-dependent manner by emodin. The potential mechanisms included were found that the emodin remarkably downregulated NF-κB DNA binding capacity, survivin, and MMP-9 in SW1990 cells. In addition, after treatment with emodin, cleaved caspase-3 expression was upregulated in SW1990 cells. Additionally, a human pancreatic cancer-induced metastatic model was created through orthotropic substitution of human tumor tissue into the pancreatic wall, and oral administration of emodin remarkably reduced tumor weight and metastasis. In addition, survivin, NF-κB, and MMP-9 expression was prevented in tumor tissues. These outcomes maintain significant information about the treatment of human pancreatic cancer using emodin as an anti-invasive agent [111]. 

Emodin has an immediate prevention on the growth effect and various malignant tumor cells metastasis. MicroRNA-1271 (miR-1271) has a comprehensive tumor suppression impact by blocking epithelial-mesenchymal transition (EMT) in tumor cells and stimulates tumor cell apoptosis. Continuing with the pancreatic carcinoma EMT regulatory mechanism, it was planned to investigate the prevention efficacy of miR-1271 and emodin against pancreatic carcinoma invasion and metastasis. EMT-related markers (E-cadherin, TWIST1, and ZEB1) expression was tested by western blot. mRNA levels of miR-1271, E-cadherin, TWIST1, and ZEB1 in pancreatic tumor cells (SW1990) were evaluated by reverse transcription-quantitative polymerase chain reaction and using Transwell tests, cell invasions was determined. Additionally, a liver metastatic model was created by implanting pancreatic tumor tissue to examine the emodin effect on the metastasis of pancreatic cancer liver. miR-1271 was presented to be remarkably reduced in pancreatic cancer cells and tissues. TWIST1 can be a target gene of miR-1271. Emodin can block the pancreatic cancer cells’ proliferation capacity and increase the expression level of miR-1271. miR-1271 remarkably restricted SW1990 cell EMT and invasive ability. In addition, emodin has been proven to inhibit SW1990 cell EMT by increasing miR-1271 levels. The data displayed that emodin stopped pancreatic cancer EMT and invasion by enhancing the miR-1271 content [112]. 

The hypoxia-inducible factor-1 (HIF-1) occurs as oxygen-sensitive HIF-1α and constitutive HIF-1β. HIF-1α cannot be detected in normal cells, but cancer cells often express HIF-1α to promote their growth, angiogenesis, and high glycolysis (also known as the effect of Warburg). The effect of Warburg in cancer cells enhances energy consumption and thus takes part in cancer cachexia, a cancer-related metabolic disorder. It was investigated to inhibit the expression of HIF-1α in human pancreatic cancer cells by emodin and rhein if any inhibitory effect alleviates cancer cachexia. Emodin and rhein reduced HIF-1α expression in MiaPaCa2 and four other human pancreatic cancer cell lines. It was also investigated the expression of HIF-1α as MiaPaCa2 cells were displayed to PX-478, noscapine, and phenethyl isothiocyanate. PX-478 and noscapine prohibited the expression of HIF-1α less than rhein and emodin, and phenethyl isothiocyanate did not show any prevention of HIF-1α expression at the concentrations tested. HIF-1α was reduced by emodin and rhein by reducing its biosynthesis, but not gene transcription or protein stability. When MiaPaCa2 cells were transferred in athymic mice, emodin and rhein stopped cancer cell growth and HIF-1α expression. They also reduced two pathological components of cancer cachexia, namely high hepatic gluconeogenesis and skeletal muscle proteolysis. As a result, emodin and rhein reduce pancreatic cancer cell growth and the expression of HIF-1α and alleviate cancer cachexia in cancer cells [113]. Dose- and time-dependant manner, the role of emodin in the treatment of pancreatic cancer has been studied on different cell lines and found to be effective. The mechanisms of action are summarized as follows: Prevention of migration and incursion, down-regulation NF-κB DNA binding capacity, survivin, and MMP-9, up-regulation cleaved caspase-3 expression; increase the expression level of miR-1271, inhibition of proliferation capacity in SW1990 cells; inhibition of the expression of HIF-1α in MiaPaCa2 cells. The in vitro effects of emodin in pancreatic cancer cell lines are summarized in Table 4.

### 3.5. Hepatocellular Carcinoma

Most of the cancer deaths in the world are caused by hepatocellular carcinoma (HCC). Although emodin has shown positive results on HCC cell lines in recent studies, its mechanism of action is still not fully elucidated. 

Zhou et al. found that 712 genes were down-regulated while 147 genes were upregulated using RNA sequencing technology in emodin-treated HepG2 cells. When the mechanisms of the identified 25 hub genes were examined, it was reported that GPR68, LPAR6, SSTR5, C5, and P2RY4 were upregulated and a significant decrease was observed in mRNA levels of these genes [114]. Emodin inhibited HCC tumor formation by upregulating miR-34a while simultaneously inhibiting ERK1/2, AKT, and VEGFR2. In addition, SMAD2 and SMAD4 expression was also suppressed. A dose-dependent decrease in mortality was observed in BALB/c nude mice treated with emodin. Cell proliferation has been shown to be stimulated by miR-34a or VEGF signal but inhibited when combined with emodin treatment [115]. It was found that emodin stimulated apoptosis in human HCC cells (Bel-7402) SREBP1-dependent and SREBP1-independent. A decrease in mitochondrial membrane potential (ΔΨm) was seen in Bel-7402 cells treated with emodin. Emodin also activated the expression of cytochrome C, caspases 9 and 3, endonuclease G, Bax, and Bcl-2 related proteins. Denaturation of fatty acids and a decrease in triglyceride levels were observed in cancer cells. It has been recorded that emodin may be effective in HCC with SREBP1 targeting [116]. In the study by Xing et al., the anti-tumoral activities of different emodin doses on HepG2 cells were investigated using ^1^H NMR complemented by flow cytometry and qRT-PCR. According to the correlation network of ^1^H NMR data and OSC-PLS-DA analysis results, an inhibition was observed on the growth of the HepG2 cells in a dose-dependent manner owing to treatment with emodin. It has also been recorded that emodin can trigger intracellular ROS formation, reduce the expression of proteins and genes involved in glycolysis, and disrupt cell cycle progression [117]. 

Sorafenib is a first-generation multikinase inhibitor and its therapeutic efficacy is reduced in advanced HCC. Therefore, combinations that increase the effectiveness of the drug are very important. Kim et al. reported that emodin significantly improves the anti-tumoral effect of sorafenib in HCC cells like PLC/PRF5, SK-HEP-1, HepG2, Huh7, and Hep3B. The transcriptional activity of SREBP-2, which suppresses cholesterol biosynthesis and AKT signal, is inhibited by emodin. It has also been observed that the AKT signal inactivates the STAT3 activator. Cell cycle arrest was enhanced by co-treatment of emodin and sorafenib in apoptotic cells and in the G1 phase. Co-treatment with emodin and sorafenib may be a potential treatment in patients with advanced HCC due to inhibition on tumor growth in SK-HEP-1 or HepG2 cells in in vivo test models [118]. HepaRG cell growth was inhibited after emodin treatment depending on time and dose and by inhibiting cell cycle progression in G2/M and S phases and stimulating apoptosis. As a result of the emodin treatment, ROS was formed that eliminates the mitochondrial membrane potential (MMP). Antioxidants such as N-acetylcysteine (NAC) suppressed these effects. Western blot analysis results showed that emodin upregulated cyclin E, p21, p53, Bax, cleaved PARP, cleaved caspase-3, 8, and 9; downregulated protein expression of Bcl-2 showed that CDK2 and cyclin A [119]. In vitro cellular mechanisms within the apoptosis pathway, uptake and cytotoxicity of HepG2 cells were investigated with GalNAc-PLGA-sTPGS nanoparticles designed to target the liver. These nanoparticles were prepared with emodin, which was proven to be effective on HepG2. The therapeutic efficacy of nanoparticles in PLC mouse model was examined. Nanoparticles were synthesized without any problem and liver targeting was achieved smoothly. Nanoparticles increased the antitumoral effect of emodin both in vivo and in vitro, performing it a possible choice for the treatment of primary liver cancer [120]. Emodin has been observed to stimulate mitochondrial dysfunction and apoptosis in HepG2 cells by upregulation of cyclophilin D. Expression of Cyclophilin D was effectively inhibited by the epidermal growth factor and N-acetyl-L-cysteine. Emodin has been reported to trigger ERK and ROS-associated Cyclophilin D expression [121]. Emodin was studied in SMMC-7721 cells at different concentrations and doses in vitroand in vivo. Emodin has been recorded to block the proliferation of SMMC-7721 cells and able to stimulate apoptosis. It has been emphasized that the possible molecular mechanisms in the realization of this event may be linked to the signaling pathways of MAPK and PI3K/AKT. While emodin was able to induce phosphorylation of ERK and p38, it suppressed AKT activation and expression of JNK. As a result of in vivo studies in mice, tumor growth was suppressed without much effect on body weight in mice. In addition, improvement was observed in the liver and kidney function of the mice [122]. The effect and underlying molecular mechanisms of emodin on the invasion and migration of MHCC-97H human HCC cells were investigated using flow cytometry and MTT assay. Emodin triggered apoptosis of cells and blocked cell proliferation in a dose and time-dependent manner. Emodin significantly (*p*< 0.05) suppressed cell invasion and migration with a treatment dose of <50 μmol/L. Also, the expression levels of MMP-2 and 9 were remarkably suppressed after emodin treatment. It has been reported that all these effects may be the result of upregulation of the MAPK signaling pathway and downregulation of the ERK/MAPK and phosphatidylinositol 3-kinase/Akt signaling pathways [122].

According to Cui et al., emodin selectively targets HepG2 cells, and the results of the antiproliferative effect were attributed to the higher ethidium bromide signal, the brighter DAPI fluorescence, the formation of ADP-ribose polymerase cleavages. Additionally, it was observed that with Annexin V-FITC/PI double staining had to measure apoptosis caused by emodin, emodin triggered higher apoptosis in comparison with the control group [123]. When the outcomes of MTT, flow cytometry, and electron microscopy performed by Zhang et al. were examined, it was observed that the antiproliferation of SMMC-7721 cells treated with 20, 40, and 80 µmol/L emodin increased after 12 h, 24 h, and 48 h depending on the time and concentration of the cells [124]. Emodin induced apoptosis in HepG2 cells through many complex events and caused significant cell accumulation in the G1 phase. According to the results of Western blot, emodin-treated HepG2 cells had enhanced release of cytochrome c from mitochondria to cytosol, up-regulated caspase-8 and 9 expression, and dose-dependent accumulations in intracellular ROS derivatives. In addition, a reduction on protein level of NF-B/p65 was observed after emodin treatment while increasing the p53 protein level in HepG2 cells. According to the computational modeling results, it was found that the emodin can be directly bound to the BH3 domain of Bcl-2. Bcl-2 expression was significantly reduced by emodin treatment, while also inhibition of heterodimerization of Bcl-2 with Bax [11]. The activity of emodin on STAT3 activation in HepG2 cells was studied in an orthotopic mouse model. When emodin was administeredintraperitoneally to mice, it inhibited the growth of hepatocellular carcinoma and STAT3 activation depending on dose and time. However, it was unable to inhibit STAT3 activation of emodin by silencing the SHP-1 gene by siRNA. After emodin treatment, the c-Src, JAK1 and JAK2 protein kinases were inhibited and a correlation was found between the inhibition of the kinases and the suppression of STAT3 [125]. Shieh et al. recorded that emodin exhibited a strong suppressive effect on PLC/PRF/5, HepG2/C3A, and SK-HEP-1 cells. Emodin stimulated apoptosis in HepG2/C3A cells due to sub-G1 accumulation and DNA fragmentation. It has been reported that HepG2/C3A cells were arrested in the G2/M phase after treatment with 60 µM emodin for 48 h. In addition, there was a significant increase in caspase-3, p53, Fas, and p21 signals. The results showed that emodin inhibited various HCC cells and caused cell cycle arrest of HepG2/C3A cells in the G2/M phase due to p53 and p21 expression. It has been recorded that apoptosis caused by emodin in HepG2/C3A cells is mediated by activation of Fas/APO-1, p53, caspase-3 and p21 [27]. Hsu et al. recorded that emodin exhibited anti-tumoral activity on HepG2, Hep3B, and Huh7 HCC cell lines with G2/M phase arrest. Western blot analysis, quantitative RT-PCR, and cDNA microarray hybridization methods were used to observe the molecular level differences. After treatment with emodin, an enhancement in the levels of Cdk2, Chk2, cyclin A, and B proteins and a reduction in the levels of p21 and Cdc25c proteins were observed in HCC cells. While RNA expression levels of CYP1A1, CYP1B1, GDF15, RASD1, MRAS, SOS1, and SERPINE1 were upregulated, TXNIP, NR1H4 and PALMD RNA expression levels were downregulated. It has been reported that these genes can be used as biomarkers for HCC treatment [126]. Emodin was observed to induce apoptosis in human HCC cell lines such as HepG2, PLC/PRF/5, and Mahlavu. After treatment of these cell lines with emodin, growth was inhibited in a time and dose-dependent manner by the production of ROS in these cells. In addition, while a decrease in intracellular ∆ψm was observed, activation of caspase-3 and -9 occurred. As a result of these events, apoptosis and DNA fragmentation occurred. The resulting oxidative stress and ROS are of great importance in the realization of apoptosis. Apoptosis was partially inhibited by preincubating HCC cell lines with cyclosporin A, H_2_O_2_ scavenging enzyme and catalase. In summary, these results show that caspase activation increased ROS formation and ∆ψ_m_ degradation after emodin treatment has a very important role in apoptosis [127]. The role of emodin in the treatment of hepatocellular carcinoma has been studied on different cell lines in vitro and in some animal experimental models in vivo and has been found to be effective. The following mechanisms of action are reported: Blockage of the proliferation, stimulation of apoptosis, induction of phosphorylation of ERK and p38, supression of AKT activation and expression of JNK; trigger intracellular ROS formation, reduction of the expression of proteins and genes involved in glycolysis, and disruption of cell cycle progression; Stimulation of mitochondrial dysfunction, stimulation of apoptosis by up-regulation of cyclophilin D, trigger ERK and ROS-associated cyclophilin D expression; enhance release of cytochrome c, up-regulation caspase-8 and 9 expression; increase in caspase-3, p53, Morocco, and p21 signals; activation of the expression of endonuclease G, Bax, and Bcl-2 related proteins; inhibition of cell cycle progression in G2/M and S phases, transcriptional activity of SREBP-2. In vitro and in vivo effects of emodin in hepatocellular cancer cell lines are summarized in Table 5.

### 3.6. Gallbladder Cancer

Gallbladder cancer (GBC) is a type of cancer that is very difficult to treat due to the resistance it develops against chemotherapeutic drugs. The development of new strategies in treatment like combination therapy is a necessity. It has been reported by Wang et al. that emodin can increase the apoptosis of gallbladder cancer cells (SGC996) stimulated by antitumor drug cisplatin (CDDP), depending on reactive oxygen species (ROS). SGC996 was treated with only CDDP or with 50 µM emodin. The viability of cells was measured by MTT assay. When CDDP was used alone at 2 μg/mL doses for 24 h, it provided ~30% suppression of viability. Emodin at 50 μM had very limited or no effect on the viability of cancer cells when used alone. However, as a result of the co-treatment of cells with this dose of emodin, an important increase was observed in the suppression of CDDP-induced cell viability. It has been observed that survivin gene expression can be suppressed by CDDP after drug treatment. As a result of the combination of emodin and CDDP, it also inhibited survivin expression due to ROS increase. In summary, emodin reduced the expression of survivin and increased the antitumor effects of in vivo CDDP, without having a toxic effect on normal tissues [128]. Emodin can play a role in reducing glutathione level in SGC996 cells and down-regulation of MDR-associated protein 1 (MRP1) expression. In tumor-bearing mice, co-treatment with emodin/CDDP was exhibited to suppress tumor growth in vivo by enhancing apoptosis of cancer cells and decreasing MRP1 expression. The concomitant treatment of emodin can significantly enhance the sensitivity of GBC compared to CDDP, oxaliplatin, or carboplatin treatment alone [129]. According to Li et al. clone formation is inhibited by emodin and effectually abolishes sphere formation of side population (SP) cells, a cancer stem cell-like cell model, and suppresses the ABCG2 function by the mechanism involved in ROS. Emodin can increase the chemosensitivity of SGC996 gallbladder cancer cells to CDDP through prevention of ABCG2 expression to defeat the resistance of SP cells at the concentrations of 20, 40, and 60 µM. Also, emodin can boost the intracellular accumulation of doxorubicin in SP cells. In vivo emodin (50 mg/kg), CDDP (2 mg/kg), and emodin/CDDP co-treatment inhibited tumor growth derived from SP cells by ABCG2 downregulating and MRP1 expression, like in vitro experiment. The results showed that emodin alone or as a complementary to chemotherapy was effective on SP cells in GBC [130].

### 3.7. Colon Cancer

Ma et al. were the first to study the apoptotic effects of emodin in the colon cancer cell line. In LS1034 cells used in in vitro experiments, 30 µm emodin caused arrest in the G2/M phase of the cell cycle and also caused a decrease in mitochondrial membrane potential and an increase in ROS production, protein levels of cyt c, caspase-9, and the ratio of Bax/Bcl-2. In the tumor model created with LS1034 cells, 40 mg/kg dose of emodin was administered *i.p* to mice. Emodin treatment resulted in a reduction in tumor weight and tumor volume [131]. Yan-Xin et al. also reported a decrease in tumor volume and weight with emodin doses applied in the range of 50–150 mg/kg in the colon cancer tumor model. The apoptosis mechanism has been associated with the inhibition of MMP-9 and VEGF-C expression [132]. It has been reported that 40 μmol/L emodin induces mitochondrial apoptosis in HCT116 cells. Increased ROS generation, overexpression of p53, therefore, up-regulated Bax expression observed after emodin treatment [133]. In LOVO colorectal cancer 0–40 µM emodin treatment resulted in decreased Bcl-2 and increased Bax levels, also increased cyt c release was observed related to the reduction in ∆Ψm [134].

Emodin showed a dose-dependent cytotoxic effect in SW480, HCT116, SNU-C2A, and SNU-C5 cells. Especially, this effect was observed more pronounced in HCT 116 cells. While HCT116 cells expressed the highest protein level of fatty acid synthase (FASN), an important enzyme for colon carcinoma development, lower protein levels of FASN were expressed in SNU-C2A, SNU-C5, and SW480 cells. Emodin (25 µM) down-regulated the protein expression of FASN, especially in HCT-116 cells, a moderate effect was observed in SNU-C5 cells, and no effect was observed in SW480 or SNU-C2A cells. The combined application of emodin with cerulenin, which is a FASN inhibitor (100 µM), resulted in an increase in apoptotic cell death compared to administration alone. In the HCT116 cells, combination treatment also caused a reduction in the PI3K/Akt phosphorylation and an increase in the ERK1/2 phosphorylation [135].

Emodin studied in the concentration range of 20–160 µmol/L in SW620 cells caused the highest apoptosis at 42.12%. It has been determined that the cell cycle is arrested in the G0/G1 phase at increasing concentrations. Decreased Bcl2, increased Bax and p53 expression have been associated with the apoptotic mechanism of emodin [136]. 

In HepG2 cells, emodin (10, 20, and 40 μM) inhibited the expression of glycolysis-related proteins. The significant increase in the percentage of the Sub-G1 phase indicates that oxidative stress due to emodin-induced ROS formation triggers apoptosis [117]. In SW480 and SW620 cells emodin (50 µM) inhibited the transcriptional activity of *β*-catenin/TCF and therefore led to a reduction in both expressions of mRNA and protein levels of *β*-catenin target genes such as c-Myc and cyclin D1 (CCD1). It was determined that the expression of the mesenchymal markers snail (SNAI1) and vimentin was also inhibited in connection with the inhibition of the Wnt signal. The resulting morphological changes indicated mesenchymal to epithelial transition in colorectal cancer cells, but alkaline phosphatase activity was induced only in SW620 cells. As shown in other studies, it was observed in this study that emodin triggers ROS and inhibits migration [137]. Activation of caspases, modulation of Bcl-2 proteins, and reduction in mitochondrial membrane potential were observed in DLD-1 and COLO 201 at doses of 18 µM and 15 µM emodin treatment. Several important components of NF-κβ, PI3K/AKT, MAPK/JNK, and STAT pathways were negatively regulated [138].

It has been proven by studies that cancer metastasis-related to epithelial-mesenchymal transition (EMT) is one of the most important causes of death of colon cancer and several signaling pathways such as RTK/Ras, Notch, NF-κB, and Wnt/*β*-catenin are associated with the activation of EMT. The study investigating the effect of emodin EMT via Wnt/*β*-catenin was started with HT 29 and RKO cells but continued with RKO cells because of the high toxicity of emodin to HT 29. MMP-7, MMP-9, and VEGF, which are marker proteins of invasion and migration were inhibited by emodin treatment at the doses of 5–20 µmol/L. An increase in E-cadherin mRNA levels and vice versa decrease in Snail, N-cadherin, and *β*-catenin expressions was also observed. Expression of the corresponding TCF4, cyclin D1, and c-Myc genes of the Wnt/β-catenin signaling pathway was downregulated. In the in vivo experimental part, in mice treated with emodin at a dose of 40 mg/kg (*p.o*), tumor growth inhibition was associated with decreased VEGF + cells, decreased expression of TCF4, cyclin D1, c-Myc, and N-cadherin, and increased expression of E-cadherin [12]. In the HCT116 colorectal cancer line, 60 µg/mL emodin inhibited VEGFR2 expression and also decreased PI3K and-AKT expression. It has also demonstrated the ability to suppress the growth, adhesion, and migration of cancer cells. In the tumor model created with HCT116 in mice, groups were treated with emodin at doses of 20, 40, 80 mg/kg (*i.p*). Decreased expression of VEGFR2, PI3K and-AKT accompanying tumor growth inhibition was observed in in vivo experiments [139]. 

In the study by Wang et al. with 20 and 40 µM doses of emodin in HCT116 and LOVO cells, it was shown that emodin induced autophagy by causing excessive localization of GFP-LC3. Increased LC3-2 accumulation and changes in p62 and Beclin-1 levels were also observed. It was determined by the cytochrome, Bcl-2, and Bax levels and ROS accumulation that emodin causes mitochondrial dysfunction [140]. 

In a different in vivo study, the antitumor activity of emodin and its analogue BTB14431 in the colon cancer model was evaluated by Höhn et al. The tumor model was formed with CC-531cells by implanting both intraperitoneal and subcutaneous, and the doses were determined as 0.3 and 1.7 mg/kg for BTB14431 and 2.5 and 5 mg/kg for emodin. Interestingly, significant results were found only at low concentrations of BTB14431, while both concentrations of emodin decreased tumor weight [141]. The SW480 cell line was used in the study to evaluate how emodin affects resistance to 5-Fu. According to the results, combined therapy (5-Fu 12 μg/mL, emodin 9 µM) reduced invasion and migration, induced apoptosis by inhibiting Bcl-2 and activating cleaved caspase3 and Bax. In in vivo experiments, 150 mg/kg of 5-Fu and 40 mg/kg of emodin were administered *i.p.* to mice both in separate groups and in combination. While tumor size was smaller in the combined group compared to other groups, the decrease in p-Akt and p-ERK expression in tumor tissues confirmed that emodin made colon cancer cells sensitive to 5-Fu [142]. In a study evaluating the effect of emodin on inflammation and tumorigenesis intestinal tumor was created with azoxymethane/dextran sodium sulfate (AOM/DSS). Emodin (50 mg/kg) treatment by gavage did not cause body weight change in the experimental mice group and reduced the incidence and size of colitis-related tumors. As a result of the examination of the tumor microenvironment, decreased inflammatory cell (CD11b+ and F4/80+) uptake, decreased expression of cytokines (IL1α/β, IL6, CCL2, CXCL5, and TNFα) and pro-inflammatory enzymes (COX-2, and NOS2) were determined [143]. In vitro biochemical effects of emodin in colon cancer cell lines are summarized in Table 6.

When the studiesare carefully examined, it is understood that the mechanism of action of emodin, which acts on cells, varies according to cell types. LS1034 cell line was arrestedin the G2/M phase and SW620 cell line was arrested in the G0/G1 phase. A suppressive effect on growth, adhesion and migration was found in most cell lines. Negatively regulated NF-κβ, PI3K/AKT, MAPK/JNK, and STAT pathways and inhibited MMP-7, MMP-9, and VEGF proteins are found to be key markers of the emodin.Like other cancer cell lines, emodin caused mitochondrial dysfunction and decreased mitochondrial membrane potential in colon cancer cell lines.

### 3.8. Cervical Cancer

Bu 25TK, Ca Ski, HeLa, and ME-180 cells were used in the first study to assess the effect of emodin on cervical cancer. Emodin treatment (50 μM) made visible many apoptotic changes in cell lines. Cervical cancer cells exhibited a significant concentration-dependent growth inhibition by emodin, but the most sensitive cell was Bu 25TK with an LD50 of 56.7 μM. Emodin has been determined to induce nuclear condensation, displacement of the phosphatidyl serine to the outer membrane surface, DNA fragmentation, and cleavage of poly (ADP-ribose) polymerase. While the activation of caspase 3 and -9 was observed, there was no change in caspase 8 activation in Bu 25TK cells [15]. Since ROS plays a key role in arsenic trioxide-induced apoptosis, emodin’s ability to increase cellular ROS levels in HeLa cells and ability to increase the apoptotic sensitivity to arsenic were investigated by Yang et al. According to the results, emodin could increase arsenic-induced apoptosis by producing ROS at a concentration of 10 µmol/L and showed no toxicity on normal fibroblast cells. Increased ROS levels were accompanied by inhibition of the activation of transcription factors NF-κB and mitochondrial transmembrane potential reduction [144]. SiHa and C33A cervical cancer cells were used in another study demonstrating emodin’s effect on increasing intracellular ROS amount and DNA damage. Also, it decreased Bcl-2 expression, and increased Bax2 in SiHa cells, and decreased AKT activation in both SiHa and C33A cells in the concentration range of 46.3–185.0 μM [145]. A study in which apoptosis and DNA damage were proven was also performed by Liu et al. Administration of emodin at doses of 1–10 μM to HeLa1.2.11 cells caused global genomic DNA damage and led to apoptosis. When applied in a dose range of 5–20 μM, S phase cell cycle arrest, and telomere damage were observed [146]. 

Treatment of HeLa cells with 40 µM emodin caused down-regulation of AKT kinase, but this effect was not associated with casein kinase (CK2) inhibition. Subsequent results of the experiment with AKT1, AKT2, and AKT3 suggested that emodin did not directly affect the activity of AKT, but instead inhibited upstream proteins that point and upregulate AKT. The application of 60 μM emodin significantly inhibited the catalytic activity of mTOR kinase by reducing the phosphorylation level of AKT protein. A slight decrease in the expression level of PTEN protein and no change in the total protein level of p44/2 MAP kinase, JNK, and p38 were also reported [147]. In a study performed by a different team on HeLa cells, emodin caused apoptotic morphological changes in the cells depending on the dose in the concentration range of 20 to 80 µM, and apoptosis was determined to be 8.2–43.7%. After 48 h of emodin treatment, mRNA expression of caspase -9, -8 and -3, cyt c, and Apaf-1 increased, FADD Fas, and FasL up-regulated, but pro-caspase-9, - 8 and -3 down-regulated. Experimental results proved that emodin induces apoptosis in HeLa cells via intrinsic mitochondrial and exogenous death receptor pathways [148]. 

A different mechanism of emodin causing apoptosis via the TGF-β signaling pathway in cervical cancer cells has been studied in SiHa and HeLa cells. It has been determined that 40 μM emodin significantly regulates the TGF-β signaling pathway by reducing the expression of P-Smad3, Smad4, and TGF-β Receptor II, and also inhibiting TGF-β induced migration and invasion [149]. 

In Hela, JAR, and HO-8910 cells, 5, 10, and 15 μM emodin exhibited antiproliferative effects and prevented migration. Increased MMP-9 mRNA expression proved the inhibition of invasion effect of emodin. Increased caspase-9 and related activation of cleaved caspase-3, DNA damage, reduction of ΔΨM, reduction of Bcl-2 was observed and all cells were arrested in the G0/G1 phase [150]. 

Emodin applied to HeLa cells in the concentration range of 1–100 μM changed the lysosomal compartment, increased autophagic vacuoles, and lysosomal hydrolase activity significantly. Increasing lysosomal membrane damage at increasing concentrations exhibited cytotoxic activity against lysosomes [151]. In their later study, Trybus et al. again discussed the apoptotic mechanism of emodin in HeLa cells in the concentration range of 1–100 μM. According to the results, it caused the highest inhibition of cell division at 100 μM, caused G2/M phase cell cycle arrest, enhanced the number of cells undergoing mitotic catastrophe, and increased changes in the cytoskeleton [152]. In vitro biochemical effects of emodin in cervical cancer cell lines are summarized in Table 7.

Although cervical cancer is the second most common malignancy in the world in terms of both incidence and mortality [148], the effect of emodin on this type of cancer has been elucidated by a limited number of studies.Considering the studies conducted, it is seen that emodin increases ROS levels, creates DNA damage and decreases the mitochondrial membrane potential.In different cervical cancer cell lines, emodin arrested the cell cycle in S phase, G0/G1 phase and G2/M phase. The activation of caspase-3 and -9, down-regulation of AKT kinase and inhibition of the catalytic activity of mTOR kinase have been revealed by different studies. Based on the findings, additional scientific evidence on the use of emodin in the treatment of colon cancer will surely be useful to fully elucidate its effectiveness.

### 3.9. Ovarian Cancer

The development of drug resistance to ovarian cancer, which causes a high numberof deaths among gynecological malignancies, is one of the reasons that precedes successful treatment. In one of the first studies conducted for this purpose, the effect of emodin on chemoresistance of A2780/taxol cells was evaluated. 1 μM paclitaxel and 10 μM emodin combination enhanced apoptosis in resistant cells compared with the alone treatment of emodin and paclitaxel. It has been stated that the apoptotic mechanism is the down-regulation of p-glycoprotein (P-gp), which increased cellular concentration of paclitaxel, and down-regulation of anti-apoptotic molecules such as X-linked inhibitor of apoptosis (XIAP) and survivin [35]. In the combined study with cisplatin, it was reported that the amount of intracellular cisplatin did not increase, contrary to the study conducted with paclitaxel because of the effect on hCtr1 influx transporter. 1 μM emodin and 100 μM cisplatin applied to the ovarian A2780 tumor cell line caused a significant reduction in both intracellular platinum levels and DNA adducts attributed to the cisplatin uptake inhibition. The results of the study emphasize that the cytotoxic effect is enhanced when protein kinase inhibitors (emodin) are treated before and not simultaneously with the chemotherapeutic agent [153]. Due to the high expression of survivin in ovarian cancers, the combined effect of survivin-targeted shRNA and emodin was evaluated in SKOV3 and HO8910 cells in a different study. A sur-shRNA plasmid was transfected into ovarian cancer cells and then the cells were treated with 60 µmol/L emodin. The administration of both emodin and shRNA caused the knockdown of survivin, therefore cell proliferation inhibition, apoptosis induction, and reduction of invasion in ovarian cells [154]. 

Based on the fact that emodin increases intracellular ROS levels, both COC1 ovarian cancer and cisplatin-resistant COC1/DDP cell lines were used in the study by Ma et al. 33 μM cisplatin and 50 μM emodin treatment increased ROS formation in resistant cells, down-regulated the multidrug resistance-related protein 1 (MRP1) gene, and induced apoptosis. In the tumor model created with COC1/DDP, both single groups of emodin (50 mg/kg) and cisplatin (1 mg/kg) and the groups in which they were combined were evaluated. According to the results of the in vivo experiments, it was determined that while no antitumor activity was observed in the group administered with emodin alone, the sensitivity of the tumor to cisplatin was increased in the group that applied combined therapy. Conjugation of nearly half of the intracellular cisplatin with GSH to form GS-platinum complexes that mask cytotoxicity and eventually extracellular transport via the glutathione conjugate export pump has also been associated with high MRP1 expression. Therefore, it was reported in the study that the amount of intracellular cisplatin increased by inhibition of MRP-1 expression [155]. In a different in vivo study, the effect of the combination of emodin (12 mg/kg) and paclitaxel (8 mg/kg) on the ovary 7,12-dimethylbenz[a]anthracene (DMBA) coated sutures were studied in rats. It was stated that the combination of emodin and paclitaxel did not cause any change in tumor size, contrary to what was reported in other studies. However, it has been interpreted that the only advantage of the combination is a reduction in the mortality rate caused by paclitaxel [156].

Emodin treatment of SK-OV-3 and A2780 cells in the 5 to 80 μM concentration range decreased the *β*-catenin, p-GSK-3β, ILK, and Slug expression. Besides, emodin has been reported to upregulate E-cadherin and claudin (epithelial markers) and downregulated N-cadherin and vimentin (mesenchymal markers). Similar effects were observed with siRNA-ILK transfection, and these effects were further enhanced after emodin (20 μM) administration, thus inhibition of epithelial-mesenchymal transition was mediated by the inhibition of the ILK/GSK-3β/Slug pathway [157]. Lu et al. subsequently created tumors in mice with SK-OV-3/pLVX-ILK or SK-OV-3/pLVX-Con cells in their in vivo study. In the treatment group, emodin (50 mg/kg/day) was administered by intraperitoneal injection every day for 21 days. In the SK-OV-3/pLVX-ILK group, the tumor was found to grow faster than the control group, SK-OV-3/pLVX-Con, but the inhibitory ability of emodin treatment on tumor growth was also determined. In vivo results supported the hypothesis that emodin inhibits proliferation by targeting ILK in ovarian cancer cells. Also, the results of the analysis of tumor tissues revealed that the E-cadherin expression was upregulated, while the Slug, MMP-9, and vimentin expressions were down-regulated [158]. Again, in A2780 and SK-OV-3 cells, Hu et al. revealed that emodin inhibited epithelial-mesenchymal transition at a concentration of 20 μM by downregulation of the GSK-3β/-catenin/ZEB1 pathway. According to the details of the study, similar to the results of Lu et al. [157], it was reported that emodin upregulates E-cadherin and keratin, while down-regulating the N-cadherin, vimentin, MMP-9, and MMP-2 [159]. In vitro biochemical effects of emodin in ovarian cancer cell lines are summarized in Table 8.

Emodin exhibited antiproliferative and apoptosis-inducing effects in ovarian cancer cells. Down-regulation of the GSK-3β/-catenin/ZEB1 pathway, N-cadherin, vimentin, P-gp, XIAP, and survivin and up-regulation of E-cadherin, claudinand keratin were shown in different studies. It has also proven to inhibit MMP-2 and MMP-9.Although the insufficiency of the studies conducted causes the mechanism not to be fully elucidated, emodin also has promising results on ovarian cancer.

### 3.10. Prostate Cancer

Despite the current clinical success that can prolong overall survival, many male patients still die due to prostate cancer. In metastatic cases, due to the primary androgen receptor (AR) signaling pathways, even if androgen deprivation (suppression) treatment is the standard treatment, it is very difficult to achieve success in treatment because of the genetic aberrations of AR. Because, within an average of 12–36 months, the majority of patients with prostate cancer develop resistance to androgen ablation, and despite testosterone, androgen-dependent / independent signals cause cancer cells to proliferate. Most of the molecular pathways involved in the initiation, development and invasion of cancer are sporadic. In this context, tumor suppressor genes and oncogenes play a role in the development and progression of cancer and the emergence of androgen-independent phenotypes. PIM1 encoding the serine/threonine PIM1 kinase is the leading oncogenes responsible for sporadic prostate cancer. DU145 cells are androgen-independent prostate cancer cells with low levels of differentiation and lack of endogenous AR expression. Emodin, a kinase blocker such as PIM kinase, has also been found to inhibit the growth of DU-145 prostate cancer cells [160]. PC3 cells, on the other hand, are low-differentiated androgen-independent prostate cancer cells that do not contain endogenous AR. Emodin inhibited the growth of these cells by stimulating the Notch signaling pathway, inducing apoptosis, and inhibiting cells in the G2/M phase [161]. Emodin was explored in previous study to act as a potent growth inhibitor in androgen-sensitive LNCaP cells provided from a male patient’s left supraclavicular lymph node, and its cytotoxic mechanism was associated with ROS production. Based on this result, it has been reported that emodin may decrease AR expression in LNCaP cells [162]. 

NF-κB is a molecule that has possible effects on carcinogenesis and the transformation of prostate cancer from an androgen-dependent to androgen-independent, untreatable form. The chemokine receptor CXCR4 is an important mediator of the interaction of prostate tumor cells with extracellular matrix proteins, supporting the invasion and migration of prostate tumor cells. Emodin inhibited NF-κB, lowered CXCR4 activation at the transcription level, thus prohibiting the invasion and migration of DU145 prostate cancer cells [69]. It has been suggested that emodin inhibits LNCaP cell proliferation by regulating the activity of AR and p53/p21 pathways, enhancing caspase-3 and 9, the Bax/Bcl-2 ratio. At this point, emodin has been found to remarkably stimulate LNCaP cell mitochondrial apoptosis compared to PC3 cells [163]. Emodin increased chemotherapeutic drugs’ cytotoxicity in prostate cancer cells and its mechanism has been linked to the prevention of multidrug resistance (MDR) and factors that cause hypoxia. After treatment with emodin plus cisplatin in DU145 cells and mouse xenografts, it has been shown to dramatically increase ROS levels, decrease MDR1 expression and decrease HIF-1 transactivation [164]. 

Hormone-resistant deterioration is an unavoidable and patients’ fatal event with advanced prostate cancer after hormone withdrawal. Increasing evidence suggests that hormone withdrawal can support this aggressive phenotype of prostate cancer. The androgen receptor (AR) in particular regulates androgen effect on tumor initiation, and behaves an important role in the relapse transition. This maintains a strong rationale for researching novel active delegates that target AR down-regulation to cure or prohibit developed the progression of prostate cancer. Emodin can immediately aim AR to prevent prostate cancer cell growth in vitro and prolong survival of C3(1)/SV40 transgenic mice in vivo. Emodin application suppressed androgen-dependent transactivation of AR by inhibiting AR nuclear translocation. Emodin reduced the relation between AR and heat shock protein 90 and increased the association of AR with the E3 ligase MDM2 (mouse double minute 2), which induces AR deterioration by proteasome-mediated route in a ligand-independent manner. Low drug toxicity and retained physical activity in C3(1)/SV40 transgenic mice caused by prostate cancer were presented by emodin [23].

Activation of p53 can stimulate p21 expression. Emodin enhanced p53 and p21 expression in LNCaP cells, thus enhancing remarkable apoptosis. Androgen receptor (AR)- and low-density lipoprotein receptor-related protein-1 (LRP1)-positive prostate cancer cell line, LNCaP is more sensitive to emodin than AR-negative LRP-positive prostate cancer cells, PC-3 cells. Low-density lipoprotein receptor-related protein-1 and androgen receptor upregulating under hypoxic conditions are involved in prostate cancer. Androgen receptor was remarkably stimulated under hypoxia-like conditions induced by CoCl_2_ and reduced with co-treatment of emodin and CoCl_2_ in AR-positive LNCaP cells. These outcomes displayed that emodin stimulates ROS-mediated growth inhibition [162]. The role of emodin in the treatment of prostate cancer has been studied and found to be effective in vitro on different cell lines (PC3, DU145, LNCaP) and in vivo C3(1)/SV40 transgenic mice models. The mechanisms of action are recorded as follows: stimulation of the Notch signaling pathway, induction of apoptosis, inhibition of the G2/M phase; inhibition of NF-B, reduction of CXCR4 activation; increase ROS levels, decrease MDR1 expression and HIF-1 transactivation; enhance p53 and p21 expression; suppression of androgen-dependent transactivation of AR, reduction of the relation between AR and heat shock protein 90, increase the association of AR with the E3 ligase MDM2.In vitro and in vivo effects of emodin in prostate cancer cell lines are also summarized in Table 9.

### 3.11. Blood System Cancer

By myeloid cell leukemia 1 (Mcl-1) elimination, cytotoxicity in human myeloma cells was remarkably stimulated by emodin. It also prohibited IL-6-stimulated Jak2 activation and the signal converter phosphorylation and transcription 3 activator (STAT3), followed by reduced expression of Mcl-1 [20]. While emodin induced caspase-3 and -9 activation, other antiapoptotic Bcl-2 family members expression other than Mcl-1 did not change in the presence of emodin. The emodin-stimulated apoptosis was almost abolished in myeloma cells overexpressing Mcl-1, close to the degree in parental cells untreated with emodin, proposing the significance of Mcl-1 in the stimulation of apoptosis by emodin. The combination of arsenic and interferon has presented suggesting outcomes to treat adult T-cell leukemia (ATL). Since the median survival of patients with ATL is about 6 months, the need for weak arsenic dose enhances in patients and the time supposed reaching a pharmacologically effective dose are major obstacles. The arsenic trioxide and interferon α (As/IFN-α) combination with emodin has a strong synergistic effect on cell cycle arrest and cell death of HTLV-I infected cells through ROS formation, and Akt and AP-1 inhibition [165]. Significantly, emodin’s practical doses allowed the concentrations of arsenic to be decreased 100-fold whilst still realizing a highly toxic effect on tumor cells. Emodin application can outcome apoptosis in human chronic myelocytic leukemia K562 cells. After emodin application, both in vivo and in vitro, the apoptosis-associated protein Bcl-2 reduced and Bax enhanced. Furthermore, activation of caspase-3, -8, and -9 by emodin has also been presented [166]. 

### 3.12. Tongue Squamous Cancer

Emodin has antitumor features such as cell cycle arrest and apoptosis in several human cancer cell lines [167]. It was presented that emodin induces apoptosis in human tongue cancer SCC-4 cells. The application of different concentrations of emodin on human tongue cancer SCC-4 cells resulted in the prevention of G2/M by supported expression of p21 and Chk2, additionally blocked cyclin B1 and cdc2, and stimulated apoptosis by the pronounced release of cytochrome c from mitochondria and activations of caspase-9 and caspase-3. The formation of reactive oxygen species (ROS), degradation of the mitochondrial membrane potency, and a reduction in the ratio of mitochondrial Bcl-2 and Bax content were determined; additionally, emodin raised the GADD153 and GRP78 levels. Free radical scavenger N-acetylcysteine and caspase blockers significantly inhibited emodin-stimulated apoptosis. These outcomes supported that emodin-mediated oxidative DNA damage depending on ROS generation and also ER stress depending on GADD153 and GRP78 levels initiate mitochondrial dysfunction from Bcl-2 and Bax modulation, mitochondrial cytochrome c release, and caspase activation, finally guiding to apoptosis in SCC-4 cells [168]. 

**Figure 6:**
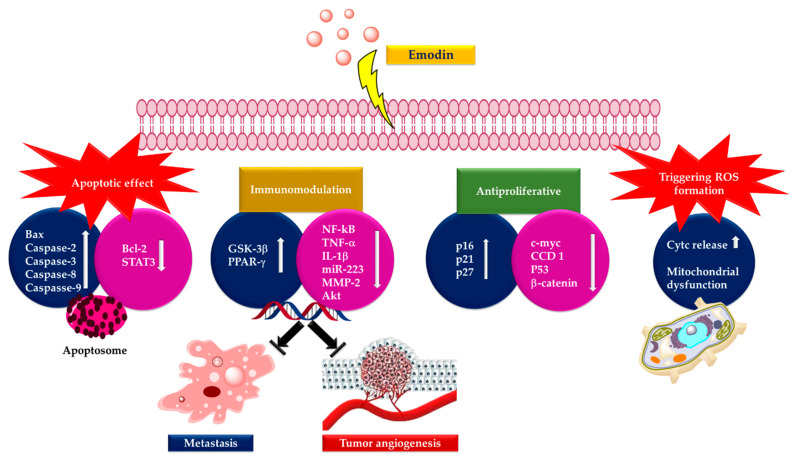
Antitumoral mechanisms of emodin.

It was also demonstrated that emodin inhibited protein levels and activities of matrix metalloproteinase-2 (MMP-2), the gene expression of MMP-9, and the migration and invasion of human tongue cancer SCC-4 cells. MMP-9 (gelatinase-B) is most commonly linked with tumor migration, invasion, and metastasis in a variety of human cancers. Emodin reduced the levels of MMP-2, urokinase plasminogen activator in a concentration-dependent manner. The molecular targeting of MMP-9 mRNA expression by emodin can be a beneficial strategy for chemoprevention and/or chemotherapeutics of tongue cancers [169]. Antitumoral mechanisms of emodin are summarized in Figure 6. 

## 4. Potential of Synthesized Derivatives of Emodin

### 4.1. Anticancer and Antiproliferative Activities

Natural products are significant structures that serve as prototypes for the development of novel medicines, particularly anticancer agents. Several anthraquinones added to this class of components were categorized as significant natural sources with these features. For example, glycosylated anthraquinone kwanzoquinone C isolated from the Kaempfer orange tree (*Hemerocallis fulva* L.) is an interesting compound for cancer chemotherapy as it has been reported to block the growth of four different types of tumor cells (GI_50_ = 3.8–7.5 µg/mL) [170,171,172]. This class of compounds has been and continues to be extensively researched due to its extensive range of biological properties. Synthetic approaches to anthraquinone skeletons are the subject of much investigation, where a few synthetic strategies have been improved to obtain this family of compounds using a variety of catalytic systems. Nowadays, most the processes displayed remarkable deficiencies like the need for multiple synthetic steps, harsh reaction conditions, and regioselectivity issues [173]. 

**Figure 7:**
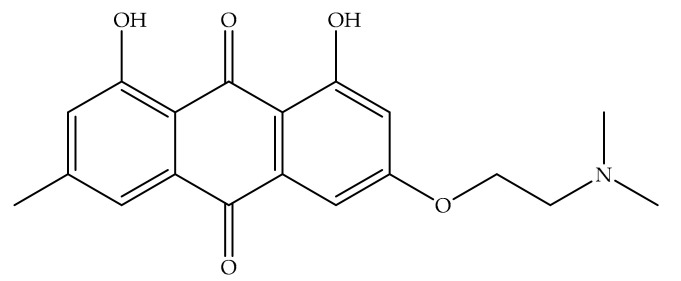
Chemical structure of compound **1**.

Great amounts of emodin can be obtained from *Rheum emodi* roots and new emodin derivatives were synthesized and their antiproliferative effects were assessed against HepG2, PC-3, DU-145, MCF-7, and HEK-293 cell lines. One of these derivatives (compound **1**, Figure 7) presented a higher in vitro anticancer activity profile, comparable to the commercial drug epirubicin. The derivative significantly blocked the proliferation of DU-145, HepG2, MCF-7, and PC-3 cancer cell lines. The derivative was also able to induce caspase-3 mediated apoptosis in HepG2 cells by halting the cell cycle in the S phase. Moreover, the derivative presented comparable DNA intercalating capacity to doxorubicin, therefore making this component an encouraging possible antitumor agent [174]. 

Emodin belongs to the same class of coplanar anthraquinones class as daunorubicin and mitoxantrone used in the clinic for more than 30 years to treat numerous types of tumors. Their function as non-covalent DNA linkers is in general believed to be crucial for their properties. However, emodin presents a low or insignificant cytotoxic effect against various cancer cells since it has low DNA binding affinity. The anthraquinone structure alone is thus not sufficient for these effects. Therefore, it is planned to improve the electron density of the system area by attaching a pyrazole ring to the anthraquinone structure by conducting synthesis studies. Thus, the compounds to be obtained should become more resistant to radical species and enzymatic degradation, reducing cardiotoxicity. The contribution of polymethyleneamine, sugar or heterocyclic side chains to the aglycone is generally important to achieve higher DNA binding affinity and antitumor effects. First, it was aimed to add a pyrazole ring to the anthraquinone structure, and then to synthesize a series of new emodin derivatives with improved DNA binding affinity and antitumor effect when various cationic amino side chains are added to this ring. DNA binding capacity of novel anthroprazoles replicated from emodin was evaluated based on interaction with calf thymus DNA. As a result, the cationic amino side chains of these compounds increased their DNA binding ability and enabled them to show strong inhibitory properties against various tumor cells. In particular, the mono-cationic amino side chains incorporated into the lead component contributed significantly to the inhibitory effects [175].

ATP citrate lyase (ACL) plays a crucial role in creating cytosolic acetyl CoA, a key building inhibitor for fatty acid and cholesterol biosynthesis. ACL is overexpressed in cancer cells and siRNA degradation of ACL limits cancer cell proliferation and decreases cancer stem. Therefore, a novel sequence of ACL blockers based on the chemical structure of natural compound emodin has been identified. A structure-activity relationship search guides the recognition of 1d as a potential lead, showing dose-dependent inhibition of proliferation and the cancer root of the A549 lung cancer cell line. This class of blockers occupies an allosteric binding site and inhibits the entry of substrate citrate into the binding site in silico, and two OH groups adjacent to the 9-carbonyl group may be crucial for activity on the goal. The halogens at the 2- and 4-position of the emodin enhanced efficiency furthest prominently, whilst the aryl substituents at the 2-position of the emodin ensued in potent ACL inhibitors. The lead components blocked the proliferation of A549 lung cancer cells in a dose-dependent manner [176].

**Figure 8:**
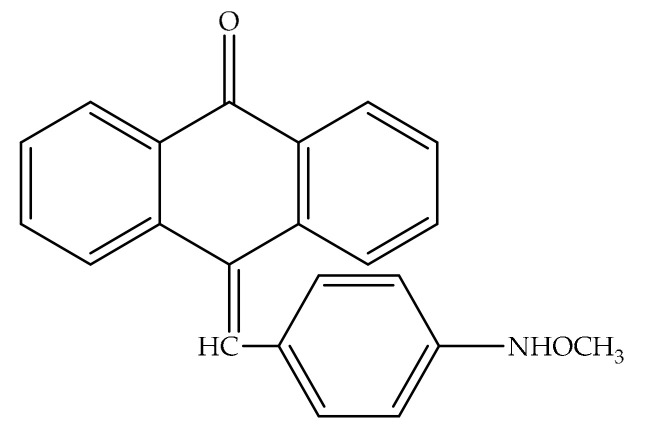
Chemical structure of DK-V-47.

Emodin prevented the HER-2/neu-encoded p185neu receptor tyrosine kinase effect in a previous work, and it was determined that a methyl, one hydroxy, and one carbonyl functional group are crucial for pharmacological properties. Therefore, nine derivatives were generated and it was found that one of the 10-(4-acetamidobenzylidene)-9-anthrone (DK-V-47, Figure 8) derivatives was more effective than emodin at suppressing p185neu tyrosine phosphorylation and preventing HER-2/neu-overexpressing human breast cancer cells’ proliferation and modification. DK-V-47 was also determined to be more effective than emodin in preventing activated HER-2/neu transformed 3T3 cells’ transformation phenotypes, containing anchorage-dependent and -independent growth, metastasis-related traits. These outcomes promote the chemotherapeutic effects of using emodin or DK-V-47 to aim HER-2/neu-overexpressing cancer cells [84]. 

Five novel emodin derivatives and their gonadotropin-releasing hormone (GnRH) conjugates were synthesized for use as possible photoactive conjugates to improve active chemotherapeutic agents aimed to malignant cells expressing the receptors. Emodin was changed at hydroxy groups and contained diverse spacers for conjugation of the peptide. Electron spin resonance and spin capture methods were used to investigate the light-induced redox features of emodin derivatives and their GnRH conjugates. Upon irradiation, all novel emodin derivatives and their conjugates induced the formation of oxygen radicals. However, remarkable variations were discovered between the derivatives tested in terms of efficiency of reactive oxygen species (ROS) generation. Due to its superior ROS generation characteristics, [D-Lys6 (MeoEmo)] GnRH was chosen as a substantial conjugate. In the way of evaluating targeting ability, this potential cytotoxic conjugate was analyzed in vitro to specify its hormonal effect and binding proximity to GnRH receptors. The outcomes showed that the binding proximity of this conjugate was comparable to that of the parent peptide. Research of in vitro LH-releasing abilities showed a relation between binding capability for GnRH receptors and bioactivity. Unification of quinones as photosensitizers to GnRH analogs could be a selective way of devastating cancerous cells owing to selective illumination of the tumor site. Breast, prostate and other types of cancers have been shown to express GnRH receptors, and it was found that GnRH analogs have exerted a direct antiproliferative activity on respective cancer cells. In fact, GnRH analogs carrying cytotoxic drugs bind with high proximity to GnRH receptors and are then adopted. Two GnRH analogs as carriers for emodin derivatives were used. The [D-Lys6]-GnRH agonist was selected since it can be selectively combined to MeoEmo, di- hydroxyEmo, trihydroxyEmo and MeoEmo-N-succinylemodin derivatives via the single free amino group at location 6 [177]. 

Many emodin derivatives (17 new ones) are attached to quaternary ammonium salts which indicated that these groups possessed two octyls or decyls were the most effective pharmacophores of all quaternary ammonium group compounds synthesized and assessed for their anticancer effects in vitro and in vivo. Components had more potent proliferative capacity against various kinds of cancer cell lines and low cytotoxicity against HELF. The compounds stimulated AGS cell apoptosis and stopped the cell cycle in a dose-dependent manner in the G0/G1 phase. Also, the activities of caspase-3, -9 enzymes were enhanced in the treated cells. In vivo studies determined that metabolites exhibited potent antitumor activity compared to the control group [178]. 

**Figure 9:**
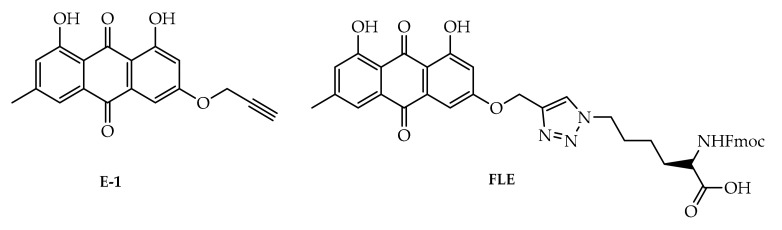
Chemical structures of synthesized emodin derivatives E-1 and FLE.

Novel anthraquinones E-1 and FLE (Figure 9) containing L-lysine groups were designed and synthesized in two steps and four steps, respectively, from emodin obtained from *Rheum ribes*. Pharmacological activity screening studies were carried out anticancer efficacy tests assessed against human colon cancer (HT-29), human cervix cancer (HeLa) and human prostate cancer (PC-3) cell lines, antioxidant effects (DPPH free radical scavenging activity, ferrous chelating capacity, and ferric reducing power assays) and interaction tests with transport protein human serum albumin. They displayed sophisticated antiproliferative effects against HeLa, HT-29, and PC-3 cell lines, whilst these derivatives presented mild to ineffectual antioxidant effects and HSA interactions. Semi-synthetic products can be potentially utilized in the improvement of novel and encouraging natural product additives [179]. 

**Figure 10:**
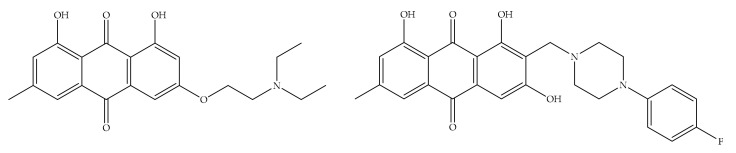
Chemical structure of new emodin derivatives I and II.

The antiproliferative efficiency of new emodin derivatives I and II (Figure 10) were synthesized and assessed against the cell lines of MDA-MB-231, HepG2, and NIH/3T3. The two derivatives significantly prevented the HepG2 and MDA-MB-231 cancer cell line proliferation, and this is similar to the drug epirubicin in the market. In addition, these metabolites were able to induce cell cycle arrest and the caspase-dependent apoptosis in HepG2 cell lines and presented DNA intercalation effect. These derivatives of emodin commit to improving safer alternatives to sold epirubicin [180]. 

A number of emodin derivatives, including 1,3-dihydroxy-6,8-dimethoxyanthra- cene-9,10-dione, have been contrived and synthesized, and 1,3-dimethoxy-5,8-dimethyl- anthracene-9,10-dione is a component that had never been reported before. These compounds exhibited neuraminidase inhibitory effect in the influenza virus with an inhibition rate of more than 50%, while four specific metabolites presented remarkable prevention of tumor cell proliferation. 1,3-Dimethoxy-5,8-dimethyltracene-9,10-dione had shown the best anticancer efficiency among all compounds synthesized by stimulating the highest rate of apoptosis in HCT116 cancer cells and stopping the G0/G1 cell cycle phase by increasing the intracellular level of reactive oxygen species. The binding of 1,3-dimethoxy-5,8-dimethyltracene-9,10-dione with the BSA protein has also been extensively studied. Therefore, this research suggested the neuraminidase inhibitory effect and antitumor activity of the novel emodin anthraquinone derivatives [181].

**Figure 11:**
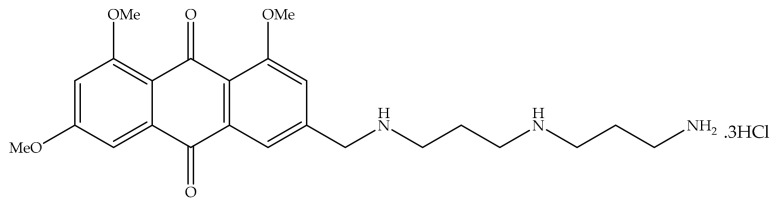
Chemical structure of potent targeted compound **6**.

A novel series of emodin derivatives was contrived and assessed for their in vitro proliferative effect. Previous records indicated that these derivatives presented weak or negligible cytotoxicity at 10 µM against several cancer cell lines (i.e., K562, HepG2, and HCT116 cell lines) as well as normal hepatic cells (QSG7701). Remarkably, appraisal for P-gp modulation showed that they have remarkable P-gp inhibitory activity. Among them, the effect of compound **6** (Figure 11) on P-gp inhibition was even better than that of the known P-gp modulator, verapamil. Consequently, the natural emodin scaffold can be used as a safe and effective modulator of P-gp mediated drug resistance in cancer chemotherapy [182]. 

### 4.2. Potential Matrix Metalloproteinases (MMPs) Activities

Compounds synthesized from emodin were assayed for their antitumor activity against the KLE cell line, a nonfunctioning estrogen receptor reproduced from undifferentiated endometrial cancer by MTT assay. The results showed that most compounds have different antitumor activity against the KLE cell line and given the presence of emodins and amines, 11 new emodin derivatives were synthesized as potential MMPIs [183]. 

### 4.3. Bone Affinity Effects

**Figure 12:**
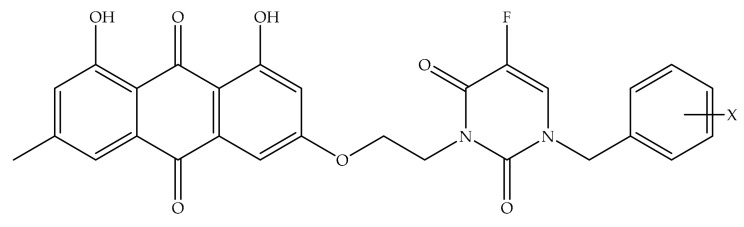
Chemical structure of emodin linked with 5-fluorouracil derivatives.

The hydroxyapative (HA) affinity test confirms that the anthraquinone components in rhubarb have a bone affinity character similar to tetracycline due to their similar structures. They all have a cyclic rigid plane structure and a hydroxyl next to the carbonyl. 5-FU is a broad-spectrum antitumor drug used in the clinic. However, it has high toxicity. The substituent group at the N atom of 5-FU has been associated with the toxicity and effects of 5-FU. In order to reduce the toxicity, emodin bound with 5-FU derivatives (Figure 12) having different substituent groups at the N atom was synthesized and their activities were investigated. Bone affinities were established by the in vitro hydroxyapatite (HA) affinity assay and their cytostatic effects were demonstrated by the MTT test by measuring the inhibition rates on osteosarcoma cells. 5-FU has low activity in vitro, it must be metabolized *in vivo* for antitumor activity. Synthetic compounds also have lower activity than 5-FU. However, they act as prodrugs of 5-FU [184]. 

### 4.4. Anti-Inflammatory Activities

**Figure 13:**
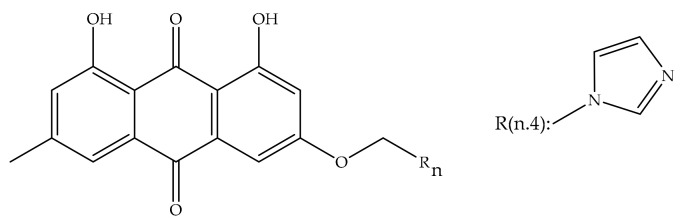
Chemical structure of some potent synthesized compounds.

Twelve azole derivatives of emodin were designed to have anti-inflammatory activity and synthesized through a two-step sequence of Williamson ether reaction and N-alkylation. The anti-inflammatory effects of these components were assessed by measuring lipopolysaccharide (LPS) stimulated nitric oxide (NO) production in RAW264.7 cells. The inclusion of imidazole and four carbons in the emodin structure administered to the detection of the active component (Figure 13), showing the best prevention of NO production among the twelve analogues. This component significantly blocks the proinflammatory cytokines interleukin-1β and -6 as well as protein and messenger RNA expressions of cyclooxygenase-2 and inducible NO synthase, and tumor necrosis factor-α in LPS-induced RAW 264.7 macrophages. This presented prevention effects on the NF-κB pathway by decreasing LPS-stimulated phosphorylation of the NF-κB blocker and nuclear translation of p-p65. These outcomes demonstrate the possible of this component in treating inflammatory cases and disorders [185]. 

## 5. Nano-Drug Delivery Strategies for Emodin

Controlled drug release has been seen as an area of increasing interest by modern drug researchers in recent years, and studies in this direction have reached satisfactory results. Controlled drug delivery systems that protect the drug create an effective, non-toxic drug concentration in the target area. Thus, broad-spectrum nano-carrier systems are frequently used in pre-clinical and clinical studies can be applied to a large number of active molecules to provide a sustained and prolonged effective concentration. In addition, improved therapeutic efficacy and bioavailability, and drug stability can be made possible by controlled drug delivery systems compared to traditional dosage regimens [186,187].

Compounds derived from plants are notable that may have a future in cancer therapy. They are found intriguing by their structure that is not toxic to normal, healthy cells, but they also produce an apoptotic response in cancer cells [188]. However, the low therapeutic index of many compounds used in cancer treatment, development of drug tolerance, damage to healthy cells during treatment and emergence of side effects are some of the problems to be overcome. 

Nano drug delivery systems are designed to alter the biodistribution and pharmacokinetics of a drug and to allow the drug to be delivered in a wider range of doses. The ability to keep the plasma level of the drug constant for a specified time is also an important advantage for controlled-release. The fact that the drug is not affected by the ambient conditions with controlled release nanosystems is a factor that increases the effectiveness of the treatment. It is also possible to increase the patient’s quality of life and compliance with these systems that target tumor tissues to improve therapeutic efficacy with increased bioavailability and reduce systemic toxicity. Enhanced targeting potential of anticancer drugs or therapeutic molecules with lipid or polymer-based nanocarriers with the use of lower doses, and controlled release are possible [189].

Emodin is known to belong to Biopharmaceutical Classification System (BCS) classification II. Owing to its weak solubility and high permeability, one of the limited bioavailability enhancement studies is nanoformulation development [190]. 

For improved oral bioavailability, Shi et al. studied the advantages of emodin nanoemulsion and its pharmacokinetics in major tissues like brain, heart, lung, liver, spleen, and kidney comparison with emodin suspension. After oral application of the nanoemulsion to rats, it effectively improved drug bioavailability and increased the mean residence time in vivo. AUC0-∞, Cmax, MRT0-∞, and t1/2 of the emodin loaded nanoemulsion were found to be 2.37, 1.62, 3.99, and 2.39 times higher than the suspension form, respectively. In particular, the 2-fold increase in the MRT of emodin in the brain than the other tissues, was found remarkable [191]. As a result, it has been shown that the nanoemulsion developed to increase the bioavailability of the emodin can effectively improve and extend the mean residence time.

One study developed nanoemulsions with spherical, nanosized droplets containing emodin to evaluate its potential in increasing transcellular absorption of emodin. It was found that increased the intracellular transit of the emodin by 2.3-fold compared to free emodin solution into UGT1A1-overexpressing Madin-Darby canine kidney (MDCKII) cells which was connected with the inhibition of uridine 50-diphosphoglucuronosyl- transferase (UGT) metabolism [192].

Solid lipid nanoparticles (SLNs) are drug delivery systems that attract attention for their high stability and excellent physicochemical and biological properties, especially for drugs with low water solubility. They were prepared to increase the anti-tumor effect of emodin by Wang et al. and technical and physicochemical properties were evaluated and characterized by particle size and distribution, zeta potential, drug entrapment efficiency, and in vitro release study. In terms of toxicity, effect on the cell cycle, and their effectiveness on human breast cancer cell line MCF-7 and MDA-MB-231 cells and cell apoptosis, the anti-cancer activity of emodin loaded SLNs was researched and found that emodin-loaded SLNs, which could be stable for 4 months and showed extended-release for 72 h, significantly increased in vitro cytotoxicity against cancer cell lines and more important cell cycle arrest effect and higher apoptotic rates in cells compared to emodin solution and empty SLNs induced [190]. 

Emodin is highly hydrophobic and soluble in organic solvents, limiting its utilization as a therapeutic agent. Therefore, liposomal emodin, both uncoated and coated with silk fibroin, was developed in order to increase its solubility and its effectiveness on Her2/neu over-expressing breast cancer cells BT-474 and MDA-MB-453 which is seen in 30% of breast cancer cells. It was found that 70% to 50% of emodin was released from uncoated and coated liposomes, respectively, with particle sizes ranging from 200 to 350 nm. In a previous study of the same study team on coating the liposome with silk fibroin, it was noted that the coating formed a tightly packed lamellar structure and slowed emodin release [193]. In the same way, emodin release was delayed with the coating in this study. and it was observed that it protected emodin against external factors and from being metabolized and was more effective in suppressing the growth of Her2/neu over-expressing breast cancer compared to uncoated emodin loaded liposomes. Although coating of emodin liposomes with silk fibroin did not change target specificity, emodin activity resulted in increased cell death with higher uptake of emodin delivered [194].

Despite the effective results of the studies mentioned above, the lack of reported *in vivo* studies draws attention. Also, PEGylation is the modification of an active molecule by linking one or more polyethylene glycol (PEG) chains that are non-toxic, non-immunogenic, and highly water-soluble, approved as a polymer by the Food and Drug Administration (FDA). PEGylation plays a significant role in drug delivery due to its innovative drug potential [195]. The pharmacokinetic parameters and bio-distribution of the drug given in the PEGylated formulation are different from the free drug and it is used in the treatment of cancer to create a specific treatment by extending circulation time in the body. In a study evaluating emodin-loaded liposomes in vivo, Wang et al., developed emodin-loaded liposomal formulation conjugated with vitamin E derivate, D-α-tocopheryl polyethylene glycol 1000 succinate (TPGS) which is a potential PEGylated alternative, compared with methoxy polyethylene glycol 2000-derivatized distearoyl phosphatidyl ethanolamine (mPEG2000-DSPE) liposomal emodin and examined in terms of biodistribution and pharmacokinetic properties.Increased cytotoxicity of emodin was found on MV4-11, K562, and L1210 leukemia cells, significant improved pharmacokinetic, and biodistribution values were observed with new, stable emodin-loaded liposomes developed with TPGS compared to mPEG2000–DSPE liposomes [196].

In breast cancer, highly invasive breast cancer cells are not completely destroyed by conventional treatments, and most survivors tends to reproduce and metastasize by forming vasculogenic mimetic (VM) channels. As a new treatment approach, the combination of targeted daunorubicin-loaded liposomes and emodin-loaded liposomes were developed by modification with arginine8-glycine-aspartic acid (R8GD) as a targeting molecule that modified liposomes surface. Destruction of VM channels and inhibition of tumor metastasis of liposomes were evaluated on MDA-MB-435S cells in vivo and in vitro by Fu et al. When we evaluate in vitro andin vivo studies together, targeted liposomes loaded with daunorubicin and emodin were accumulated in the tumor area with the effect of enhanced permeability and retention (EPR). An increased absorption was also observed, modification of the surface of liposomes was also improved cellular uptake and targeting and the formation of VM channels and metastasis are inhibited [197]. It has been stated that the combination of liposomes can be used as an effective treatment method with the synergistic effect of daunorubicin with antitumor effect and emodin, which has been proven effective against cancer by many studies. 

Transfersomes are drug delivery systems that increase skin penetration and have been extensively studied in recent years. Due to their flexible structure and similarity to the biological membrane, they effectively increase the therapeutic effectiveness of the drug [198]. In addition, nanotransfersomes developed for other administration routes are also available in the literature. One of these studies is investigating the anti-cancer effects of the combined use of a novel nano carrier as a nanoemodin transfersomes and ultrasound in head and neck squamous cell carcinoma (HNSCC cell lines). Hemolytic activity, cell proliferation, apoptosis and mRNA expressions of caspase 3 and 9 assayed to evaluate the anti-cancer effect of novel nano carrier on cancer cells. Considering all the results, thanks to this new carrier system, which increases its effect with its sound sensitizing aspect, the anticancer effect of emodin increased on HNSCC cell lines by causing decreased cell viability and induced apoptosis significantly [199]. 

Previously specified as an alternative to PEGylation process, the water-solublevitamin E derivative TPGS, is used in many studies as a solubility and absorption enhancer, emulsifier, plasticizer, and carrier for water-insoluble and fat-soluble drugs system. In one of them, Liu et al. formulated polylactic-co-glycolic acid (PLGA), which is a biocompatible and biodegradable polymer, commonly used in the pharmaceutical field, and TPGS nanoparticles containing emodin, investigated the effect of formulations in liver cancer both in vivo andin vitro, and aimed to increase the efficiency with multiple targeted nanoparticles developed by forming a complex polymer matrix of PLGA-TGPS compared with only PLGA nanoparticles. Increased cellular uptake by HepG2 and HCa-F showed that PLGA-TPGS nanoparticles could be absorbed efficiently. An increase in the rate of apoptosis cells was monitored with the formulations administered in a dose-dependent manner. In vivo studies, nanoparticles had a longer half-life and mean retention time than emodin solution with an obvious sustained-release and PLGA-TPGS nanoparticles reflected its antitumor effect more clearly was obtained than emodin-PLGA nanoparticles [200]. The importance of specific targeting has once again been proven with these results. The presence of TGPS allowed for easier internalization of liver cancer cells with drug and played a better role against cancers [201]. In addition, TGPS increased the permeability, in vivo uptake, and efficacy of drugs in accordance with the literature.

In similar study, the aim was to achieve active targeting to the liver with N-acetylaminogalactosyl-decorated biodegradable PLGA-TPGS copolymer nanoparticles including emodin. The in vitro cytotoxicity, cellular uptake, mechanism, and apoptosis of HepG2 cells and the in vivo therapeutic effects of nanoparticles were analyzed at primary liver cancer mouse model. In addition to the effective results of PLGA-TPGS copolymer nanoparticles, which were previously developed to improve water solubility, which is limiting in the therapeutic efficacy of emodin, in this study the active targeting provider molecule structure, D-α-N-acetylaminogalactosyl (GalNAc) was linked to developed PLGA-TPGS copolymer nanoparticles. Based on the high affinity of GalNAc for the highly expressed asialoglycoprotein receptors on the surface of liver parenchymal cells, the new GalNAc-PLGA-TGPS biomaterial that could be actively targeted to the liver was synthesized. As a result, GalNAc-PLGA-TGPS nanoparticles exhibited a stronger antiproliferative effect on the HepG2 cell compared to the free emodin. Better active targeting and higher levels of inhibition of liver cancer cells were observed in mice [120]. It can be said that the newly synthesized GalNAc-PLGA-TPGS biomaterial has an important role in active targeting with the increase in asialoglycoprotein receptor expression.

In another study, Wang et al. succeeded in encapsulating the polyphenolic-structured emodin into uniform nanoparticles functionalized with phenylboronic acid. All parameters and processes in formulation development were standardized and nanoparticles were characterized in terms of morphology, particle size, polydispersity, loading efficiency. Cytotoxicity test carried out with MTT test and findings showed that developed new nanoparticles were effective and promising due to the higher cytotoxicity on HepG2 cancer cells compared to the healthy MC-3T3-E1 cells [202].

Utilizing a combination of targetability and sustained release properties, polylactic acid (PLA) microspheres containing emodin were developed and characterized in vitro and the therapeutic efficacy evaluated in mice with lung fibrosis by Chen et al. In this research, it was observed that the pathological changes in the heart, liver, spleen or kidney, which were mentioned in previous studies, due to the frequent use of emodin, were not found in the tissues of animals administrated with PLA microspheres [203]. These lung-targeted microspheres are thought to have therapeutic potential in intensive lung cancer, avoiding off-target toxicities previously reported for emodin.

In the literature it was reported that mesoporous silica-based nanoparticles can be used for many potent antitumor molecules in controlled drug release systems with improved drug adsorption features and pre-calculated release kinetics [204]. In one study conducted with the first use of amino-functionalized mesoporous silica for water-insoluble emodin by Xu et al. They performed release experiments in phosphate-buffered saline (pH 7.4) to evaluate the release profile of emodin from the mesoporous matrices and it was found that surface amino-functional transporters were found to be able to control the emodin release rate [187]. 

Mesoporous silica slows down its rapid release by protecting emodin in the extremely acidic environment of the stomach also protects emodin against light-induced degradation. In a similar study, stability and cytotoxicity of emodin loaded mesoporous silica (E-MS) was investigated as a vehicle for the transport through tumor cell lines (human melanoma A375 and mouse melanoma B16, B16F10). In cytotoxicity studies performed at different doses, the dose-dependent decrease in cell viability was found to be directly related to the emodin content and tumor cell growth was inhibited by E-MS. Their findings suggested that the solubility and stability of the emodin was provided with the developed E-MS system, as well as efficient cellular uptake of the compound. The solubility and stability of the emodin was improved, as well as efficient cellular uptake of the compound with the developed silica system. Conversely, the retention effect and enhanced permeability are achieved by the selective penetration into the tumor tissue and is retained within the cells [204]. 

The activity of emodin with known anticancer effects is limited by its poor solubility and lack of specific targeting, reducing its bioavailability. Magnetic nanocarrier systems are widely used, especially for targeting purposes, for example magnetic resonance imaging (MRI) diagnosis and in magnetism-induced drug delivery. To enhance the chemotherapeutic effect of emodin compared with free emodin and to improve bioavailability, Song et al. developed the emodin-loaded magnetic liposomal formulation containing ferromagnetic iron oxide nanocubes and assessed the activity of formulation in vivo andin vitro. A further 8.67% increase in killing efficiency was obtained on the MCF-7 breast cancer cell line by magnetic targeting compared to free emodin. Besides, this magnetic formulation was evaluated in 4T1 tumor-bearing mice. Compared to the control and free emodin groups, 16 times faster tumor growth and 12 times more tumor weight were observed at the end of 14 days in the magnetic liposomal emodin applied groups. After administration of the therapeutically effective magnetic emodin liposomes, whose biocompatibility was confirmed by hemolysis, cytotoxicity, and biochemistry tests, a reduction in tumor growth was found significantly [205].

## 6. Toxicity of Emodin

Interest in natural products is increasing day by day, but in most cases, clinical data and toxicological surveys of such preparations are lacking. In the EU, there are several medicinal products based on emodin-containing herbal drugs on the market. Since the laxative effect of preparations based on anthraquinone-comprising laxatives is applied by disturbing the epithelial cells that can conduct to the improvement of colorectal carcinoma, it is recommended to use such preparations for a short time only in case of occasional constipation. In the prospective case-control study of colonoscopy patients, it was found no link between the use of anthraquinone-containing laxatives and colorectal carcinoma. More recent Italian surveillance on reports of suspected adverse reactions associated with anthraquinone-containing laxatives presented that such preparations should be used only for a limited period of time [206]. 

Emodin has been recorded to cause genotoxicity, nephrotoxicity, hepatotoxicity, and reproductive toxicity. Emodin caused considerable apoptosis in a time-dependent manner as specified by morphological changes in L-02 cells. Additionally, emodin has found to be the possible to disrupt glutathione and fatty acid metabolism in human liver cells. The US National Toxicology Program has reported that exposure to emodin resulted in enhanced cases of renal tubule pigmentation in male and female mice and increased cases of nephropathy in female mice. Administration of emodin ensued in a remarkable reduction in cell viability and inhibition of the proliferation of HK-2 cells [207,208]. Emodin has been recorded to cause reproductive toxicity. Using mouse blastocysts as an experimental model, it was studied whether the mammalian apoptotic inducing emodin has cytotoxic activities on embryonic development. The efficacy of emodin on in vitro developmental damage of blastocysts and in vivo embryo transfer were also studied. With this study, it was noted that emodin induced apoptosis in the inner cell mass and trophectoderm of mouse blastocysts, guiding to a reduction in embryonic development and viability. The input showed that emodin is a damage risk factor for normal embryonic improvement [209]. In male reproductive tissues/organs, a toxicity survey of emodin has shown that it has testicular toxicity due to impaired expression of testicular genes. Emodin also inhibits human sperm functions by decreasing sperm [Ca2+]i and suppressing tyrosine phosphorylation. *R. patientia* ethanol extracts, which include anthraquinones like chrysophanol and emodin as effective compounds, created irreversible pathological alterations at very high doses (4000 mg/kg), but lower doses and water extracts were manufactured with no important or reversible alterations. Thus, prolonged application of high doses of anthraquinone extracts during pregnancy should be completely refrained [208,210]. 

The mechanism of the side effects of emodin is complex and not fully understood at this time. The toxicity of emodin was planned to specify a cell metabolomic technique to study and explore the toxicity, possible mechanism, and related aims of the emodin. Metabolomic profiles of cell extracts and cell culture media acquired using 1H-NMR analysis were utilized to evaluate emodin toxicity in HepG2 cells. Multivariate statistical analyzes like partial/orthogonal partial least-squares-discriminator tests were used to define metabolites that varied between control and emodin groups. Emodin caused variations in 33 metabolites, including creatine, acetate, aspartate, arginine, histidine, leucine, and isoleucine in cell extract samples, and glutamate, alanine, isoleucine, formate, and succinate and in cell culture medium samples. Nearly eight pathways linked with these compounds in the emodin groups were impaired. These outcomes indicated the possible of utilizing cell metabolomic approaches to elucidate the toxicological effects of emodin, underlying mechanisms, and active biomarkers. These results could aim to the improvement of new strategies to explore goals for drug toxicity, explain the alterations regulatory signaling networks, and find possible mechanisms of activity [211]. 

## 7. Conclusions

Emodin has been suggested to be effective for cancer management, principally in digestive system cancers (like pancreatic cancer) by modulating multiple molecular targets included in tumor growth, angiogenesis, invasion, and metastasis according to in vivo or in vitro experimental models. In current years’ investigations, emodin was shown to have anticancer activity in different cancer types, however, the mechanisms of different cancer types seem to alter. Indorsing mitochondrial apoptosis might be a comparable emodin’s antitumor mechanism in various cancer types.

Many researchers have previously conducted studies on the activity of emodin on various tumor cells and reported its antitumor effects. Despite these studies, the specific anticancer mechanisms of emodin have not been fully elucidated. The recent resurgence of interest in research on the anticancer effects of emodin in the last 5–10 years has made great progress in evaluating the effects of this compound. Emodin has been found to act on different cancer types through different mechanisms. Therefore, more studies are required to define the exact antitumor effect mechanisms of emodin in each case.

Another issue is that emodin displays toxicity, including hepatotoxicity, nephrotoxicity, genotoxicity, and reproductive toxicity, which appear to be contradictory. On the one hand, reports on side effects are also increasing. Based on the literature review, the reasons for these effects are likely to be related to the dose and duration of administration. High doses and prolonged administration may cause these toxicities. Emodin shows hepatotoxicity and nephrotoxicity by inducing apoptosis in the caspase 3-dependent pathway and in the mitochondrial pathway. Although there have been attempts to eliminate of minimize these effects by preparing synthetic emodin derivatives, more studies are needed to reduce the toxicity and at the same time to eliminate these contradictions when it comes to the phrase “two sides of the coin”.

Based on these comments concerning the effects of emodin, these results may provide evidence that can be utilized in the design strategy of new therapeutic agents that block tumor cells. Notwithstanding the hopeful outcomes abstracted here, more studies regarding the antitumor mechanisms of emodin and clinical trials are necessary and compulsory to approve the clinical antitumor activity previously the opportunity of large-scale clinical administration. In spite of the fact that traditional medicines investigation has been significantly performed with the advent of novel techniques, studies are needed to obtain stronger evidence confirming the clinical applications of herbal medicines.

In addition to biological activity studies of plants, their contents should be determined, standardized (to be sustainable) and posology studies should be done in order to be used in clinical studies as mentioned. There are also several studies on qualitative and quantitative analysis of the major compounds included in the quality studies for herbal drugs carrying anthraquinone derivatives (especially emodin). Anthraquinone and anthraquinone glycosides were obtained from the dried rhizomes of *Polygonum cuspidatum* Sieb using HPLC-PAD-MS method for qualitative and quantitative analysis of major compounds. For this study, five Rhizoma Polygoni Cuspidati samples taken from different regions were analyzed using the developed method. The validation studies of the method have been carried out, and since the analytical procedure is precise and reproducible, it has been found suitable for the analysis of a large number of samples. It was planned to create an index for quality assessment in using the plant as a standardized herbal drug and piceid, resveratrol, emodin-8-*O*-glucoside and emodin were determined as the main components [212].HPLC-PAD-MS method was used for qualitative and quantitative analysis of five different *Polygonum multiflorum* Thunb samples obtained from different regions of China by the same study group, similarly, nine compounds including stilbene glucosides, anthraquinone glucosides and anthraquinone derivatives were identified. Repeatability was evaluated with intra-day and inter-day tests, and recovery studies were performed. The results show that there is a change in the contents of the components found in samples of the same plant obtained from different regions. Such variations are presumably attributable to differences in growing opr cultivation conditions. Although the compound amounts vary, the analytical procedure developed has been shown to be precise and reproducible. This method has been found suitable for fast and easy analysis of anthraquinone derivatives [213]. With five samples from each plant, *Rheum officinale*Baill. (RO), *P. multiflorum* Thunb. (PM) and *P. reynoutria* Makino (PR) were analyzed simultaneously with the HPLC-DAD-MS method on the samples prepared by a Dionex pressurized liquid extraction system from the roots of the plants, and the characteristic compounds were identified. A comprehensive validation of the developed method has been made and good sensitivity, repeatability and accuracy data are presented. Fourteen components were identified by comparison with the online ESI/MS and literature data and standard compounds. It is seen that the contents of PR, PM and RO samples collected from various regions are composed of compounds with the same chemical structure (stilbens and anthraquinones). The stilbene group components of the three plants differed more than the anthraquinones in terms of majority. Emodin is the anthraquinone compound commonly found in all three herbs, but is different in content. The average anthraquinone abundance in RO is higher than PR or PM, and the variation in anthraquinone content between PR and PM is not very significant. This result revealed that PR and PM have a close relationship (may be plants of the genus *Polygonum* in the Polygonaceae family), but that RO is far from PR or PM in plant taxonomy. Therefore, emodin, one of the characteristic components, can be selected as an analytical marker for quality assessment and chemical verification in plants belonging to these families. The results also showed that this analytical method has good precision, accuracy and sensitivity that help control the quality and safety of these herbs [214].

In order to support all these investigations and not be misleading in biological activity/cytotoxicity studies, several studies have been carried out in an effort to obtain higher yields of emodin from plants and to increase its use as an active ingredient of appropriate purity in the treatment. Li et al. investigated how to increase the extraction yield of emodin from *P. cuspidatum* by enzymolysis methods. The yield of emodin was increased by cellulose degradation before extraction, and the optimum enzymolysis conditions were: (1) cellulase dosage 2 mg/g, (2) pH: 3.0, (3) temperature: 40 °C, and (4) enzymolysis time: 1.0 h. Extraction after enzymolysis was carried out using 60% ethanol with a solid/liquid ratio of 1:20 at 50 °C for 2.0 h. The extraction yield of emodin was determined as 0.78%. Compared with the common extraction method, it has been observed that the enzymolysis method can increase the extraction yield of effective ingredients such as emodine [215].

Emodin-8-*β*-glucoside from *Polygonum cuspidatum* Sieb et Zucc was transformed into emodin by fermentation using *Rhizopus microsporus*. The fermentation products were purified by various column chromatographies (H1020 resin, silica gel). Thin layer chromatography and high performance liquid chromatography were used to identify the compounds and evaluate the transformation efficiency. In particular, the change of *β*-glucosidase activity and various fermentation parameters were monitored simultaneously, the 0.4 g/L value given for emodin at the beginning reached 0.65 g/L in 80 h. The transformation rate of glycosides was 98% and the purity of emodin was 95% [216].

## Figures and Tables

**Table 1:** Summary of biochemical effects of emodin in lung cancer cell lines.

Cell Line	Cell Type	Concentration	Mechanism	Reference
A549	Lung adenocarcinoma	50 µM	Increased cyt c, activation of caspase-2, -3, -9, and mitochondrial Bax Inactivation of ERK and AKT, formation of ROS, disruption of ∆Ψm, reduction of mitochondrial Bcl-2	[14]
CH27	Lung squamous cell carcinoma	50 µM	Morphological change, sub-G1 formation, disruption of focal adhesion kinase Increased expression of Bak and Bax proteins, activation of caspase-3, -8, -9	[25]
CH27	Lung squamous cell carcinoma	50 µM	Internucleosomal DNA fragmentation Increase in cyt c, activation of caspase-3, expression of PKCa Decreased expression of PKCδ and ε	[58]
H460	Non-small human lung carcinoma			
NCI-H446	Small cell lung cancer	20 μmol/L	Increased caspase-3 activation Up-regulated NACA, p8, and PQBP1 genes Downregulated B2M, HLA-E, and CD1D genes	[62]
A549 H1650	Lung adenocarcinoma	2–10 μM (Emodin) 2 μM (Gefitinib)	Triggered Rad51 protein instability Decrease in phospho-ERK1/2 and Rad51 protein levels	[63]
A549	Lung adenocarcinoma	50 µM	Activation of the ATM-p53-Bax signaling pathway, ROS formation	[64]
H1650 A549	Lung adenocarcinoma	25–100 µM	Inactivation of ERK1/2 Down-regulation of Rad51 and ERCC1	[65]
H520	Human bronchioloalveolar cell carcinoma			
H1703	Lung squamous cell carcinoma			
H1650 A549	Lung adenocarcinoma	25–200 µM (capecitabine) 50 µM (emodin)	Inactivation of ERK1/2 Down-regulation of Rad51 and ERCC1 Increased mRNA and protein expression of TP	[66]
H520	Human bronchioloalveolar cell carcinoma			
H1703	Lung squamous cell carcinoma			
H1703 A549	Lung squamous cell carcinoma Lung adenocarcinoma	1 µg/mL (cisplatin) 8.1; 16.2; 24.3 µg/mL (emodin)	Instability of the ERCC1 protein Inactivation of ERK1/2	[67]
SK-MES	Lung squamous cell carcinoma	40 μmol/L (SK-MES) 70 μmol/L (A549)	Decreased expression of ERCC1 and Rad51 protein and mRNA	[68]
A549	Lung adenocarcinoma			
A549	Lung adenocarcinoma	100 μM	Decreased expression of CXCR4 and HER2	[69]
A549 PC9	Lung adenocarcinoma	50 μM	Increase in the phosphorylation of AMPKα and ERK1/2 Decrease in the expression of ILK	[71]
H1299 H1650 H1975	Non-small cell lung carcinoma			
A549	Lung adenocarcinoma	5 to 50 μM (emodin) 50 to 2000 nM (daunorubicin)	Inhibition of anthracycline reductase enzymes Sensitized cells to daunorubicin	[70]
A549	Lung adenocarcinoma	50 μM	Arrest in G2/M phase Invasion inhibition Increased PPARγ protein and luciferase reporter activity	[72]
H1975	Non-small cell lung carcinoma			
A549	Lung adenocarcinoma	1 nM to 10 µM	Prevention of ATP-induced increases in P2X7 Invasion inhibition	[73]
A549 H1299	Lung adenocarcinoma	80 µmol/L	Increased caspase 3 activation, TRIB3 expression	[74]
A549	Lung adenocarcinoma	30 to 100 µM/mL	Arrest in G1 and G2/M phase Loss of ∆Ψm Increased release of cyt c	[75]
A549	Lung adenocarcinoma	10,15, and 20 µM	Inhibition of p53 protein aggregates	[76]
A549	Lung adenocarcinoma	Emodin 10 μM Paclitaxel 4 μM	Increased Bax and active caspase 3 expressions Decreased Bcl-2, p-Akt, and p-ERK levels	[77]
A549	Lung adenocarcinoma	5 µM emodin, 5–10 µM cisplatin (A549) 2.5 µM emodin, 5–10 µM cisplatin (H460)	Increased DNA damage Decreased Pgp expression	[79]
H460	Non-small human lung carcinoma			
NCI-H-520	Human bronchioloalveolar cell carcinoma	50 µM	Inhibition of MTH1, arrest in G2/M Increased expression of Bax, survivin, P-21, cleaved caspase 3, cleaved PARP (A549) Decreased Bcl-2, p-65 NFκβ (A549) Reduced integrin β1 and vimentin protein expression (A549)	[81]
NCI-H-460	Non-small human lung carcinoma			
A-549	Lung adenocarcinoma			
H1703	Lung squamous cell carcinoma	60 μM	Decreased levels of mitomycin C-derived Rad51 mRNA and protein	[82]
A549	Lung adenocarcinoma			
A549	Lung adenocarcinoma	30 µM	Suppressed the secretion of HA Augmented cells in G1/G0 phase and reduced cells in S and G2/M phase (A549) Decreased cyclin A and B, increased cyclin C,D,E (A549)	[83]
H520	Human bronchioloalveolar cell carcinoma			
H1975	Non-small cell lung carcinoma			
H1299	Lung adenocarcinoma			
H460	Non-small human lung carcinoma			

**Table 2:** Summary of in vitro biochemical effects of emodin in breast cancer cell lines.

Cell Line	Concentration	Mechanism	Reference
MDA-MB453 BT-483 AU-565	40 µM	Suppressing the autophosphorylation and transphosphorylation activities of HER-2/neu tyrosine kinase	[17]
MDA-MB453 MCF-7 B104-1-1	40 µM emodin 20 µM potent derivative: 10-(4-acetamidobenzylidene)-9-anthrone)	Suppressing the tyrosine phosphorylation of p185^neu^, inhibiting the proliferation and transformation of HER-2/neu-overexpressing human breast cancer cells Cell cycle arrest at G0/G1 phase in MDA-MB 453	[84]
MDA-MB-231 MCF-7	110 µM emodin 5 µM doxorubicin	Increased γH2Ax expression and the DNA damage Decreased expression of the AKT1, p53, PARP1, RAD51, and XRCC1 signaling pathway	[80]
ZR-75-1 MCF-7	60 µM emodin 4 µM tamoxifen	Up-regulation of cyclin D1 and p-ERK	[85]
MDA-MB-231	10–40 µM	Inhibition of TPA-stimulated MMP-9 activity Reduced transcriptional activity of AP-1 and NF-kB	[86]
BCap-37	50 µM	Increased the percentage of cells in the sub-G0/1 phase Decreased Bcl-2, Increased Bax level, and cytosolic cyt c level	[87]
BCap-37	50 µM	Up-regulation of P21 Down-regulation of IGF-2 Induced gene expression of p53 and caspase 3	[88]
SKBR3	25 and 50 μM	Increased caspase 3, 8, and 9 mRNA levels Increased Bax level Reduced Bcl 2 level	[89]
MDA-MB-453 MCF-7/ADR MCF-7	2.3 to 9.2 µg/mL emodin azide methyl anthraquinone derivative	Lowered the Her2/neu protein Inhibited the downstream MAPK and PI3K-Akt signaling pathway by inhibiting p-Akt, and p-ERK1/2 in the MDA-MB-453	[90]
MDA-MB-453		Arrested the cell cylce in the G0/G1 phase Inhibited the expressions of: Cyclin D1, c/Myc, CDK4, and p-Rb	[91]
MCF-7/Adr	20 μg/mL	DecreasedERCC1 expression	[92]
MCF-7 MDA-MB-453	25 μM	Effect on nuclear ERα distribution	[93]
MCF-7	35 µM	Single-stranded DNA breaking, DNA fragmentation, up-regulation of FASL gene expression Down-regulation of the expression of CCND1, C-MYC, and MCL1	[94]
MDA-MB-435s	1 to 10 µM	Inhibition of ATP-induced increase in [Ca2+]	[73]
MCF-7	20 and 40 µM	Arrested the cell cycle in the G0/G1 phase Blocking the effect of estrogen on ERα expression and transcriptional activity Down-regulation of cyclin D1 and Bcl-2 protein expression, decreased PI3K/Akt protein expression	[95]
EO771-GFP	10 µM and 30 µM	Reduced adhesion between macrophages and cancer cells	[99]
4T1	100 µM	Decreased macrophage migration	[97]
EO771 4T1	0 to 50 μmol/L	Inhibition of the adherence of macrophages to the monolayer of tumor cells	[100]
4T1	25 µM	Decreased TGF- β1 production Inhibited the formation of EMT and CSC	[101]
MDA-MB-231 MDA-MB-453	25 μM	Inhibition ofcell migration and invasion Decreased CCL5 levels Inhibition of the phosphorylation of AKT, activation of GSK3β, downregulation of the expression of β-catenin, Vimentin and snail Increased expression ofE-cadherin	[102]
MCF-7	20 μM emodin 40 μM 5-FU	Up-regulation of p21, p16, p27 protein Down-regulation of E2F1 and NRARP protein	[103]
MCF-10A MCF-7 MDA-MB-231	Berberine + emodin (5, 10, 20 μM)	Suppressed SIK3 activity Cell cycle arrest (G0/G1)	[104]
MCF-7 T47D	Emodin 10 µg/mL and thymoquinone 2 µg/mL	Cell arrest in the sub G0/G1 phase, an increase in p53, Bax, and cleaved caspase 3 expression levels, a decrease in Bcl-2 protein, induction of ROS formation, and Cyt C releasein MCF7 cells Inhibition of cell migration and FAK, pFAK, and integrin β1 proteins were down-regulated in both cell lines	[105]
MCF7	25–100 µmol/L	Increase in CYP1A1 expression Regulating the expression of AhR and CYP1A1 proteins	[106]

**Table 3:** Summary of in vitro biochemical effects of emodin in gastric cancer cell lines.

Cell Line	Concentration and/or Method of Application	Efficacy and Mechanism	Reference
MKN45	0.05 mM emodin	Inhibition of proliferation Arrest in the G0/G1 and G2/M phase	[107]
SNU-5	Combination with cisplatin (25 μM emodin + 3.0 μM CDDP)	Increase the apoptotic effect on cells and cell cycle arrest	[108]
SGC-7901	Emodin (10 μM)	Down-regulation of PRL-3 mRNA	[109]
SGC-7901	Emodin-loaded nanomicelles	Cytotoxic effect	[77]
BGC823 MGC803	Emodin loaded stearic acid-chitosan oligosaccharide	Antitumoral effect	[110]

**Table 4:** Summary of in vitro biochemical effects of emodin in pancreatic cancer cell lines.

Cell Line	Concentration	Mechanism	Reference
SW1990	Dose-dependent manner (10, 20, 40 µmol/L)	Prevention of migration and incursion Down-regulation NF-κB DNA binding capacity, survivin, and MMP-9 Up-regulation cleaved caspase-3 expression	[111]
SW1990	Dose- and time-dependent manner (0, 20, 40 µmol/L)	Increase the expression level of miR-1271 Block proliferation capacity	[112]
MiaPaCa2	Dose-dependent manner (Varied from 50 μM to 100 μM)	Inhibition of the expression of HIF-1α	[113]

**Table 5:** Summary of in vitro and in vivo biochemical effects of emodin in hepatocellular cancer cell lines.

In Vitro (Cell Line)/ In Vivo Studies	Concentration and/or Method of Application	Mechanism	Reference
SMMC-7721	Dose-dependent manner (<50 µmol/L emodin)	Block the proliferation Stimulation of apoptosis Induction of phosphorylation of ERK and p38 Supression of AKT activation and expression of JNK	[122]
SMMC-7721	20, 40, and 80 µmol/L	Antiproliferation activity	[124]
HepG2	Different emodin doses (79.01, 51.39, 33.13 µM)	Trigger intracellular ROS formation, reduction of the expression of proteins and genes involved in glycolysis, and disruption of cell cycle progression	[117]
HepG2	Dose-dependent manner (50, 100 µM emodin)	Stimulation of mitochondrial dysfunction Stimulation of apoptosis by up-regulation of cyclophilin D Trigger ERK and ROS-associated cyclophilin D expression	[121]
HepG2	120 µM emodin	Induction of apoptosis and cause cell accumulation in the G1 phase Enhance release of cytochrome c Up-regulation caspase-8 and 9 expressions	[11]
PLC/PRF/5, HepG2/C3A, SK-HEP-1	60 µM emodin	Stimulation of apoptosis Arrest in the G2/M phase Increase in caspase-3, p53, Fas, and p21 signals	[27]
HCC (Bel-7402) with SREBP1 targeting	100 μmol/Lemodin	Stimulation of apoptosis Decrease in mitochondrial membrane potential Activation of the expression of cytochrome C, caspases 9 and 3, endonuclease G, Bax, and Bcl-2 related proteins	[116]
HCC (PLC/PRF5, SK-HEP-1, HepG2, Huh7, and Hep3B)	Emodin and sorafenib	Inhibition of transcriptional activity of SREBP-2 Stimulation of cell cycle arrest in G1 phase	[118]
HepaRG	80 µM emodin	Inhibition of cell cycle progression in G2/M and S phases Stimulation apoptosis Up-regulation of cyclin E, p21, p53, Bax, cleaved PARP, cleaved caspase-3, 8, and 9 Down-regulation of protein expression of Bcl-2	[119]
HepG2/PLC mouse model	GalNAc-PLGA-sTPGS nanoparticles with emodin	Increase the antitumoral effect	[120]
BALB/c nude mice model	Dose-dependent manner (1 mg/kg or 10 mg/kg emodin)	Up-regulation miR-34a Inhibition of ERK1/2, AKT, and VEGFR2 Supression of SMAD2 and SMAD4 expression	[115]
HepG2 /Orthotopic mouse model	Dose-dependent manner (max. inhibition occurring at around 50 μM)	Inhibition of c-Src, JAK1 and JAK2 protein kinases	[125]

**Table 6:** Summary of in vitro biochemical effects of emodin in colon cancer cell lines.

Cell Line	Concentration	Mechanism	Reference
LS1034	30 µM	Cell cycle arrest in the G2/M phase Decrease in ∆Ψm Increase in ROS production, protein levels of cyt c, caspase-9, and the ratio of Bax/Bcl-2	[131]
HCT116	40 μmol/L	Increased ROS generation, overexpression of p53, up-regulated Bax expression	[133]
LOVO	0–40 µM	Decreased Bcl-2 and increased Bax levels Increased cyt c release, reduction in ∆Ψm	[135]
HCT-116	25 µM	Decreased protein levels of FASN	[135]
HCT-116	25 µM emodin 100 µM cerulenin	Increase in apoptotic cell death and ERK1/2 phosphorylation Reduction in the PI3K/Akt phosphorylation	[135]
SW620	20–160 µmol/L	Cell cycle arrest in the G0/G1 phase Decreased Bcl2, increased Bax and p53 expression	[136]
HepG2	10, 20, and 40 μM	Inhibition of the expression of glycolysis-related proteins Increase in the percentage of the Sub-G1 phase	[117]
SW480 SW620	50 µM	Inhibition of the transcriptional activity of *β*-catenin/TCF Reduction inc-Myc and CCD1 levels Inhibited SNAI1 and vimentin levels Inhibition of the Wnt signal	[137]
DLD-1 COLO 201	18 µM 15 µM	Negatively regulated NF-κβ, PI3K/AKT, MAPK/JNK, and STAT pathways Activation of caspases, modulation of Bcl-2 proteins, and reduction in ∆Ψm	[138]
RKO	5–20 µmol/L	Inhibited MMP-7, MMP-9, and VEGF proteins Increase in E-cadherin mRNA levels Decrease in Snail, N-cadherin, and *β*-catenin expressions Downregulated TCF4, cyclin D1, and c-Myc genes	[12]
HCT116	60 mg/mL	Inhibition of VEGFR2 expression Decreased PI3K and-AKT expression Suppressive effect on the growth, adhesion, and migration	[139]
HCT116 LOVO	20 and 40 µM	Increased LC3-2 accumulation and changes in p62 and Beclin-1 levels Mitochondrial dysfunction	[140]
SW480	12 μg/mL 5-Fu 9 µM emodin	Reduced invasion and migration Inhibiting Bcl-2 and activating cleaved caspase-3 and Bax	[141]

**Table 7:** Summary of in vitrobiochemical effects of emodin in cervical cancer cell lines.

Cell Line	Concentration	Mechanism	Reference
Bu 25TK	56.7 μM	Activation of caspase-3 and -9 Induction of nuclear condensation, DNA fragmentation, and cleavage of poly (ADP-ribose) polymerase	[15]
HeLa	10 µmol/L	Increased ROS levels Activation of transcription factors NF-κB Reduction in ∆Ψm	[144]
SiHa C33A	46.3–185.0 μM	Increased intracellular ROS and DNA damage Decreased Bcl-2 expression, and increased Bax2 in SiHa cells, and decreased AKT activation in both SiHa and C33A cells	[145]
HeLa1.2.11	1–10 μM 5–20 μM	Genomic DNA damage S phase cell cycle arrest, and telomere damage	[146]
HeLa	40 µM 60 μM	Down-regulation of AKT kinase Inhibition of the catalytic activity of mTOR kinase Reduction in the phosphorylation level of AKT protein	[147]
HeLa	20 to 80 µM	Increased mRNA expression of caspase-9, -8 and -3, cyt c, and Apaf-1 Up-regulated FADD Fas, and FasL Down-regulatedpro-caspase-9, -8 and -3	[148]
SiHa HeLa	40 μM	Reduction in the expression of P-Smad3, Smad4, and TGF-β Receptor II Inhibition of TGF-β induced migration and invasion	[149]
Hela JAR HO-8910	5, 10, and 15 μM	Increased MMP-9 mRNA expression Increased caspase-9 and related activation of cleaved caspase-3, DNA damage, reduction of ΔΨM, reduction of Bcl-2 Cell cycle arrest in the G0/G1 phase	[150]
HeLa	1–100 μM	Increased lysosomal membrane damage	[151]
HeLa	1–100 μM	G2/M phase cell cycle arrest Enhanced mitotic catastrophe	[152]

**Table 8:** Summary of in vitro biochemical effects of emodin in ovarian cancer cell lines.

Cell Line	Concentration	Mechanism	Reference
A2780	1 μM paclitaxel 10 μM emodin	Down-regulation P-gp, XIAP and survivin	[35]
A2780	1 μM emodin 100 μM cisplatin	Reduction in both intracellular platinum levels and DNA adducts	[153]
Sur-shRNA plasmid transfected SKOV3 and HO8910	60 µmol/L	Knockdown of survivin Proliferation inhibition, apoptosis induction, and reduction of invasion	[154]
COC1 cisplatin-resistant COC1/DDP	33 μM cisplatin 50 μM emodin	Increased ROS formation Down-regulatedMRP1 gene Induction of apoptosis	[155]
SK-OV-3 A2780	5 to 80 μM	Decreased the β-catenin, p-GSK-3β, ILK, and Slug expression Up-regulation of E-cadherin and claudin Downregulation of N-cadherin and vimentin	[157]
A2780 SK-OV-3	20 μM	Down-regulation of the GSK-3β/-catenin/ZEB1 pathway Up-regulation of E-cadherin and keratin Down-regulation of N-cadherin, vimentin, MMP-9, and MMP-2	[159]

**Table 9:** Summary of in vitro and in vivo biochemical effects of emodin in prostate cancer cell lines.

In Vitro (Cell Line)/ In Vivo Studies	Concentration and/or Method of Application	Efficacy and Mechanism	Reference
PC3	125, 250, 500 μg/mL emodin	Stimulation of the Notch signaling pathway Induction of apoptosis Inhibition of the G2/M phase	[161]
DU145	100 μM emodin	Inhibition of NF-κB Reduction of CXCR4 activation	[69]
DU145	Emodin plus cisplatin	Increase ROS levels Decrease MDR1 expression and HIF-1 transactivation	[164]
LNCaP	Dose-dependent manner (Maximum at 40 µmol/L emodin)	Enhance p53 and p21 expression Stimulation of ROS-mediated growth inhibition	[162]
C3(1)/SV40 transgenic mice model	Dose-dependent manner (Low-10 μmol/L and high-40 μmol/L concentration)	Suppression of androgen-dependent transactivation of AR Reduction of the relation between AR and heat shock protein 90 Increase the association of AR with the E3 ligase MDM2	[23]
